# Multimorbidity among midlife women in India: well-being beyond reproductive age

**DOI:** 10.1186/s12905-022-01693-2

**Published:** 2022-04-12

**Authors:** Parul Puri, Abhinav Sinha, Pranab Mahapatra, Sanghamitra Pati

**Affiliations:** 1 Department of Survey Research and Data Analytics, International Institute for Population Sciences, Mumbai, Maharashtra India; 2 Health Technology Assessment in India (HTAIn), ICMR-Regional Medical Research Centre, Bhubaneswar, 751023 Odisha India; 3 Department of Psychiatry, Kalinga Institute of Medical Sciences, Bhubaneswar, Odisha India; 4 ICMR-Regional Medical Research Centre, Chandrasekharpur, Bhubaneswar, Odisha India 751023

**Keywords:** Chronic diseases, India, Midlife, Multimorbidity, Women, LASI

## Abstract

**Background:**

Currently, inequality in life expectancy across gender makes women outlive men. Adult women transit towards menopause around midlife accompanied by a series of natural physiological changes leading to several conditions such as osteoporosis, depression, and urinary incontinence, which puts them at a higher risk of having multimorbidity. Multimorbidity is often associated with poorer quality of life, leading to deteriorated work productivity and associated economic loss in midlife. Hence, this study aimed to determine the magnitude and correlates of early onset of multimorbidity and explore its linkages with health-related quality of life (HRQoL) among middle-aged women in India.

**Methods:**

We have utilized data from the first round of the Longitudinal Ageing Study in India, 2017–19. We included women aged 45–65 years (n = 23,951) for analysis. Descriptive data were presented. An ordered logistic regression was conducted and proportional odds were reported to identify the correlates of multimorbidity. To explore the linkages between multimorbidity and selected indicators of HRQoL, an array of regression models were executed.

**Results:**

Multimorbidity was reported amongst 29.8% of women in midlife. Chandigarh (PR-54.8 PER 100 women) and Punjab (PR-52.8 per 100 women) reported the highest prevalence of multimorbidity. Women with multimorbidity reported compromised HRQoL indicators such as self-rated health, work-limiting health conditions, mobility, and activities of daily living.

**Conclusions:**

Multimorbidity is increasingly prevalent in midlife women associated with inferior quality of life. The reproductive health programs for women should consist of midlife women focusing on multimorbidity and overall well-being.

**Supplementary Information:**

The online version contains supplementary material available at 10.1186/s12905-022-01693-2.

## Background

Ageing is an inevitable natural process often linked with declining health conditions [1]. Healthy ageing does not have a universally laid criterion but can broadly be regarded as maintaining robust physical, mental, and social health, leading to overall well-being [2]. The demographic and epidemiological transition in low-and-middle-income countries (LMICs) has led to the rise in co-existing two or more long-term conditions known as multimorbidity [3, 4]. Projections suggest adults aged 45 years and above will constitute over 655 million or 40 percent of the Indian population by 2050 [5]. The burden of multimorbidity often increases with a rise in age, which is evident by the findings of our previous study to assess multimorbidity amongst adults in primary care settings of Odisha, India, which revealed prevalence ranged from 5.8% in participants aged 18 to 29 years to 45% among those aged more than 70 years [6].

India witnessed a dramatic increase in life expectancy from 42.27 years in 1960 to 69.16 years in 2017 due to healthcare technology and quality advancements [7]. Currently, inequality in life expectancy across gender makes women outlive men [7]. Women in LMICs like India fall under the vulnerable and disadvantaged section of society due to socio-economic and cultural barriers, hindering their comprehensive development. Further, this implies that a mere increase in life expectancy does not guarantee a healthy life.

According to the World Health Organization (WHO), gender is an essential factor in determining health outcomes in an individual [8]. Women seldom have a say in their decision-making process, including their health [9]. Adult women transit towards menopause around 45 years; this is accompanied by a series of natural physiological changes [10]. These physiological changes compel them to be more aware of their health and healthy ageing at mid-life. Also, a change in health and consumption behaviours is generally observed around the pre-retirement age.

Broadly, the focus on women’s health remains confined to sexual and reproductive health, with almost no importance garnered to post-menopausal or health at mid-life. Reproductive, Maternal, Neonatal, Child and Adolescent Health (RMNCH + A) program under National Health Mission (NHM) is a strategy to promote interventions throughout lifecycle approach but does not cover health beyond reproductive age. Around midlife, women may develop several conditions such as osteoporosis, depression, and urinary incontinence due to menopausal transition [11]. In India, chronic non-communicable diseases (NCDs) typically start a decade earlier (around 45 years and older) than in high income countries [12]. Multimorbidity steeply rises in middle-aged adults with the accumulation of different chronic conditions, which plateaus among the elderly. A systematic review to find the relationship between multimorbidity and health-related quality of life (HRQoL) showed a higher prevalence of multimorbidity in midlife studies than studies reporting the adult population [13]. It also revealed HRQoL to be poorer amongst midlife participants in both studies reporting only midlife and the entire adult population [13]. Multimorbidity, in general, is associated with increased healthcare utilization and expenditure, poorer quality of life, and psychological distress needing greater attention in resource constraint settings [14]. This calls for comprehensive evidence on multimorbidity at midlife, especially in LMICs like India, where such data is scarce, with no such studies reported among women.

Midlife represents a critical period of health transition requiring optimal health attention. This escalates among women due to the socio-economic and cultural barriers and their physiological needs, which require intensive interventions to combat. Poorer quality of life in midlife can deteriorate work productivity and associated economic loss. Hence, this study was conducted to inform healthcare professionals and policymakers with evidence on multimorbidity among women at midlife, based on the nationally representative data from Longitudinal Ageing Study in India (LASI) wave-1. The specific aim was to determine the magnitude and correlates of early onset of multimorbidity and explore its linkages with health-related quality of life (HRQoL) among middle-aged women (45–65 years) in India.

## Methods

### Data

The present study utilized data on 23,951 women aged 45–65 years (middle-aged) from the first wave of the Longitudinal Ageing Study in India (LASI), 2017–18. LASI was conducted as a joint venture by the International Institute for Population Sciences (IIPS), Harvard T. H. Chan School of Public Health (HSPH), and University of Southern California (USC); and was launched under the stewardship of the Government of India [15]. LASI wave-1 provided nationally representative information on men and women in the age range of 45 years and above and their respective spouses who resided in the same household, irrespective of age [15]. LASI employed a multi-staged stratified area probability cluster sampling design. Additional information on the survey design and sampling procedure can be found on the website of IIPS, Mumbai [15].

For the present study, two separate datasets, namely individual (n = 72,250) and biomarker (n = 65,900) were employed. These datasets were merged and information on 65,900 individuals were acquired. In view of the study’s objective, information specifically on women in the age-group 45–65 years was required. For this, from the merged dataset information on 43,412 individuals in the age-group 45–65 years was extracted, moreover, we obtained information on 23,951 women for the final analysis.

### Ethics statement

LASI received ethical approval from the Indian Council of Medical Research (ICMR), New Delhi and the International Institute for Population Sciences (IIPS), Mumbai. At the unit level, individuals were supplied with a catalogue containing the information on the purpose of the survey, confidentiality, and safety of health assessment. Written consent forms were administered at household and individual levels, in accordance with the Human Subject Protection. In totality, LASI administered four separate written consent forms, i.e., household, individual, consent for blood samples collection for storage and future use, and proxy consent [15].

### Study variables

Present study examined a multidimensional linkage between socio-economic and demographic [16, 17], health behaviours [18, 19], anthropometric predictors [20, 21], and family and reproductive predictors [22–24] with single and multimorbidity [25]. Socio-economic and demographic context included information on the respondent’s age (in years), residence, religion, social group, level of education, occupation, and wealth. Health behaviours included consumption of tobacco, ever used alcohol and physical activity. The waist-hip ratio was included as an anthropometric variable. Family and reproductive variables included marital status, parity, family history of chronic disease, living arrangements, and experienced menopause.

Considering the missing values, except for two variables, namely ‘occupation’ and ‘experienced menopause’ no missing values were present in the dataset. For occupation, 2263 women had not specified their category. Similarly, 1368 (5.1%) of the women did not know about their menopause status. Although there was no way to draw relevant inferences for these categories, dropping these observations would have added a biasedness in the data estimates. For the same reason, we added an additional category ‘not classified’ and ‘don’t know’ for occupation and experienced menopause respectively.

Information on seventeen self-reported chronic non-communicable diseases were used to generate a chronic disease score (CDS). These diseases included asthma, musculoskeletal disorders, cancer, chronic bronchitis, chronic renal disease, chronic obstructive pulmonary disease (COPD), diabetes mellitus, gastrointestinal disorders, chronic heart disease, high cholesterol, hypertension, neurological and psychiatric disorders, obesity, skin disorder, stroke, and thyroid disorder and urinary incontinence. CDS was further segregated into three sub-groups: Group 1: No morbidity (women with no chronic NCD), Group 2: Single morbidity (women with exactly one chronic NCD), and Group 3: Multimorbidity (women who are affected with two or more chronic NCDs simultaneously) and used as an outcome of interest.

Further, the study examined the linkages of multimorbidity with selected indicators of HRQoL. LASI did not collect direct information on HRQoL, thus, the study employed six proxy indicators, namely self-rated health (SRH), work limiting health conditions, mobility restrictions, Activities of Daily Living (ADL), Instrumental Activities of Daily Living (IADL) and Life Satisfaction to measure HRQoL among middle aged women in India. A detailed description on the indicators of HRQoL is presented in Table 1.

**Table 1:** Description of the indicators of health-related quality of life (HRQoL) included in the study, Longitudinal Ageing Study in India (LASI), 2017–18

Indicators	Question	Method [categories]
Self-rated health	In general, would you say your health is excellent, very good, good, fair and poor?	Recoding into ordered categories [good, fair, poor]
Work limiting health conditions	Do you have any impairment or health problem that limits the kind or amount of paid work you can do?	Recoding in binary categories [no, yes]
Mobility	Do you have difficulty with…..? 1. Walking 100 yards (yes/no) 2. Sitting for 2 h or more (yes/no) 3. Getting up from chair after sitting for long period (yes/no) 4. Climbing one flight of stairs without resting (yes/no) 5. Stooping, kneeling or crouching 6. Reaching or extending arms above shoulder level (either arm) (yes/no) 7. Pulling or pushing large objects 8. Lifting or carrying weights over five kilos, like a heavy bag of groceries (yes/no) 9. Picking up a coin from a table (yes/no)	Scoring Method + Recoding into binary categories [none, at least one]
Activities of daily living (ADL)	Because of your health or memory problem, do you have any difficulty with…? 1. Dressing, including putting on chappals, shoes, etc. (yes/no) 2. Walking across a room (yes/no) 3. Bathing (yes/no) 4. Eating difficulties (yes/no) 5. Getting in or out of bed (yes/no) 6. Using the toilet, including getting up and down (yes/no)	ADL score Count Variable (range 0–6)
Instrumental activities of daily living (IADL)	Because of your health or memory problem, do you have any difficulty with…? 1. Preparing a hot meal (cooking and serving) (yes/no) 2. Shopping for groceries (yes/no) 3. Making telephone calls (yes/no) 4. Taking medications (yes/no) 5. Doing work around the house or garden (yes/no) 6. Managing money, such as paying bills and keeping track of expenses (yes/no) 7. Getting around or finding address in unfamiliar place (yes/no)	IADL score Count Variable (range 0–7)
Life satisfaction	Please say how much do you strongly agree, slightly agree, neither agree nor disagree, slightly disagree, somewhat disagree or strongly disagree with the following statements 1. In most ways my life is close to ideal 2. The conditions in my life are excellent 3. I am satisfied with my life 4. So far, I have got the important things I want in life 5. If I could live my life again, I would change almost nothing	Categories of all the five questions were made unidirectional. Internal consistency was checked using Cronbach’s alpha. Further, the scores were generated using a multiple correspondence analysis. These scores were recategorized to form Life satisfaction with three ordered categories namely (Low, Medium, High)

### Statistical analysis

We used descriptive statistics, including mean, standard deviation, range, frequencies and weighted percentages to provide the background of the study population. Disease profile was illustrated using prevalence (per 100 women) calculated as:

$$\begin{aligned} & {\text{Prevalence}}\;\left( {{\text{per}}\;100\;{\text{women}}} \right) \\ & = \frac{{{\text{All}}\;{\text{new}}\;{\text{and}}\;{\text{existing}}\;{\text{cases}}\;{\text{during}}\;{\text{a}}\;{\text{given}}\;{\text{time}}\;{\text{period}}}}{{{\text{Surveyed}}\;{\text{individuals}}\;{\text{during}}\;{\text{the}}\;{\text{same}}\;{\text{time}}\;{\text{period}}}} *100 \\ \end{aligned}$$

Further the distribution of Chronic Disease Score (CDS) with three categories viz. no morbidity, single morbidity and multimorbidity was presented over age. In addition, sub-national level variation in the distribution of single and multimorbidity was also illustrated.

A Variance Inflation Factor (VIF) was used to assess multicollinearity between the selected predictors before executing the final model. The primary outcome of this study was CDS, which comprised of three ordered categories, viz no morbidity, single morbidity and multimorbidity. A bivariate ordered logistic regression model was utilized to assess the association between multimorbidity and background characteristics; unadjusted odds ratios along with 95% confidence intervals were reported. A multivariable ordered logistic regression analysis was conducted to identify the correlates of multimorbidity among middle-aged women in India.

In addition, we examined whether multimorbidity (simultaneous occurrence of two or more NCDs) has an adverse implication on the health-related quality of life (HRQoL) as compared to single morbidity (base outcome). For this, linkages of six proxy variables were studied with the chronic disease score.

For association between CDS (none/single (base outcome)/multimorbidity) with self-rated health and life satisfaction, two separate multivariable ordered logistic regression models were executed. At the same time, two separate multivariable binary logistic models were performed for work-limiting health conditions and mobility restrictions. Adjusted Odds Ratios were reported along with 95% Confidence Intervals (CI) for the four aforementioned HRQoL indicators.

As activities of daily living (score range 0–6) and instrumental activities of daily living (score range 0–7) were two count variables with frequencies inflated at zero, we utilized a zero-inflated Poisson’ (ZIP) regression model and reported prevalence rate ratios with 95% CI. The suitability of ZIP model over the traditional Poisson’ regression model was assessed using Vuong’s test (a significant z-test value was reported in both the cases). All the multivariable models were adjusted for selected demographic health behaviours, anthropometric predictors, family, reproductive variables and sub-national level (state) variations included in the analysis.

Statistical analysis and data visualization was performed with STATA version 15.0 (StataCorp™, Texas) and RStudio version 1.1.463 (R Studio, Inc.). A *p*-value < 0.05 was considered to be statistically significant for all calculations. All estimates were reported by applying appropriate sampling weights [15].

## Results

### Description of the study population

This study is based on 23,951 women in the age-range 45–65 years from LASI, wave-1, 2017–18. Table 2 gives the description of the study population. The mean age the women surveyed was 54.4 years. Around seventy percent lived in rural areas. Eighty-two percent of the individual followed Hinduism and forty-five percent belonged to the other backward classes. Around sixty-two percent of the women received no education and 61.3 percent were unemployed. Around 42 percent belonged to deprived class.

**Table 2:** Descriptive statistics for women in mid-life (45–65 years), Longitudinal Ageing Study in India (LASI), wave-1, 2017–18

Correlates	Mean (Standard Deviation)	(Maximum, Minimum)
*Continuous variables*		
Age (in years)	54.4 (6.29)	(65, 45)
Parity	3.76 (2.22)	(22, 0)
Waist-hip ratio	0.92 (0.08)	(1.8, 0.26)

Correlates	Frequency (N = 23,951)	Weighted percentage
Categorical variables		
*Residence*		
Rural	15,501	69.00
Urban	8450	31.00
*Religion*		
Hindu	17,514	81.85
Muslim	2978	11.36
Christian	2363	3.43
Others	1096	3.37
*Social group*		
Scheduled castes	4150	20.02
Scheduled tribes	4301	8.96
Other backward class	8995	45.13
Other castes	6505	25.89
*Level of education*		
No education	13,587	62.20
Less than primary	2387	8.89
Primary completed	2959	10.77
Middle completed	1940	6.58
Matric completed	1585	4.97
Intermediate complete	689	3.46
Above intermediate	804	3.14
*Occupation*		
Unemployed	14,957	61.30
Blue collar	5754	27.62
White collar	417	1.24
Pink collar	560	2.27
Not classified/others	2263	7.58
*Wealth*		
Deprived class	9480	41.97
Affluent class	14,471	58.03
*Consumption of tobacco*		
Tobacco abstainer	19,501	82.06
Only smoking	785	2.78
Only smokeless	3545	14.83
Both smoke and smokeless tobacco	120	0.33
*Ever use alcohol*		
No	22,993	97.36
Yes	958	2.64
*Physical activity*		
Physically active	18,604	78.34
Physically inactive	5347	21.66
*Waist-hip ratio*		
Low risk	4464	21.68
High risk	19,489	78.32
*Marital status*		
Currently in union	17,725	74.49
Not in union	6226	25.51
*Family history of chronic disease*		
No	14,493	61.75
Yes	9458	38.25
*Living arrangements*		
Living alone or with others	1708	7.33
Living with family members	22,243	92.67
*Experienced menopause*		
No	2285	6.98
Yes	20,298	87.92
Don't know	1368	5.10

With respect to health behaviors, 82 percent never consumed tobacco, 97.4 percent were lifetime alcohol abstainer, and 21.7 percent were leading a physically inactive lifestyle. Furthermore, on an average the middle-aged women had a parity of four children and WHR of 0.92 (standard deviation = 0.08).

Around seventy-five percent of the women were currently in union. Thirty-eight percent had a family history of chronic diseases. Eighty-eight percent had experienced menopause.

### Burden of selected non-communicable diseases and multimorbidity

**Fig. 1:**
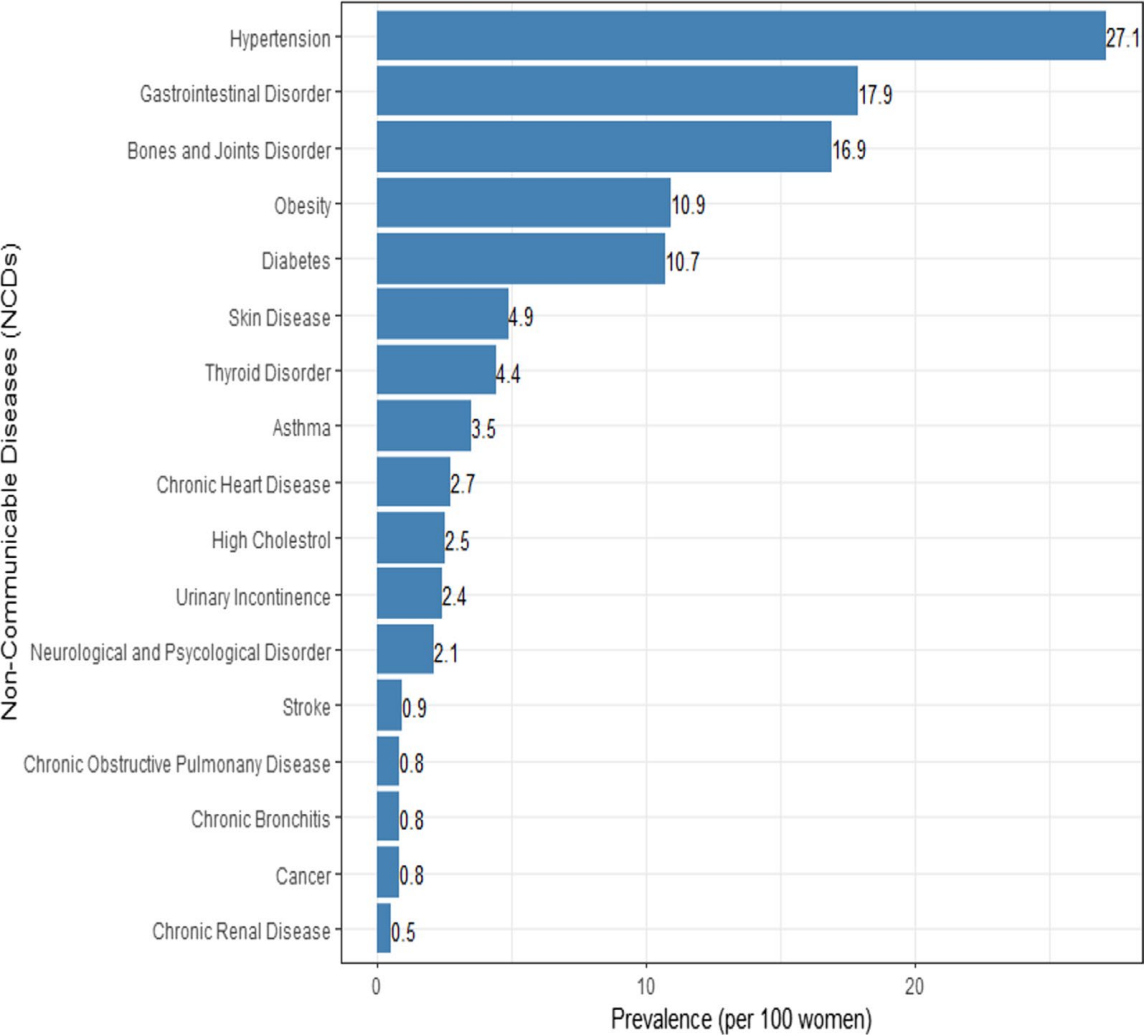
Prevalence of selected non-communicable diseases among women in mid-life (45–65 years), Longitudinal Ageing Study in India (LASI), wave-1, 2017–18

Figure 1 further illustrates the distribution of selected NCDs among middle aged women in India. The findings suggest that hypertension (Prevalence = 27.1 per 100 women), gastrointestinal disorder (Prevalence = 17.9 per 100 women), musculoskeletal disorder (Prevalence = 16.9 per 100 women), diabetes (Prevalence = 10.7 per 100 women), and obesity (Prevalence = 10.9 per 100 women) were most commonly occurring NCDs.

**Fig. 2:**
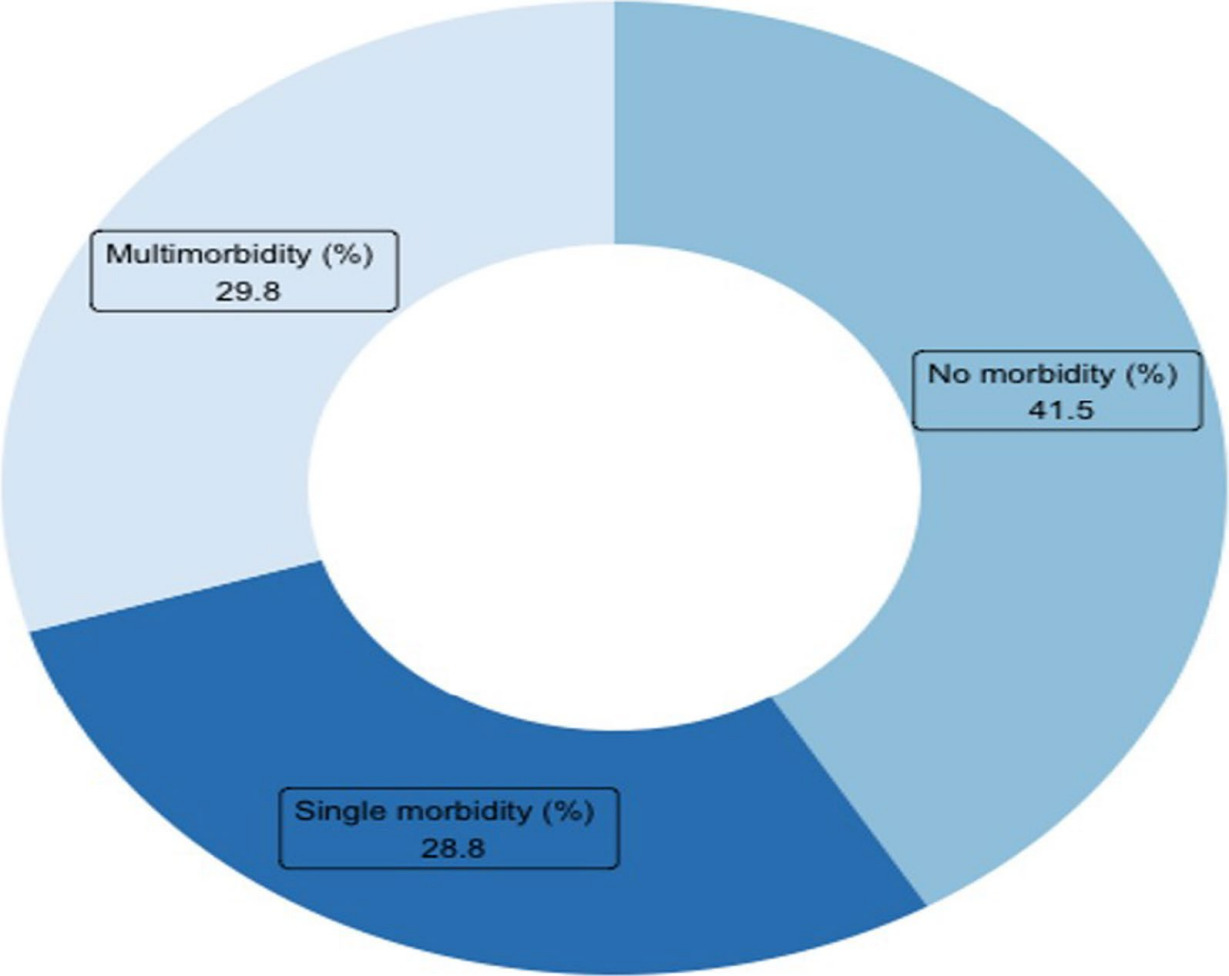
Prevalence of none, single and multimorbidity among women in mid-life (45–65 years), Longitudinal Ageing Study in India (LASI), wave-1, 2017–18

The findings from Fig. 2 suggest that 41.5% of the women had no NCDs, while 28.8 and 29.8 percent had single or multimorbidity, respectively.

### Variation in the prevalence of single and multimorbidity by age-groups

**Fig. 3:**
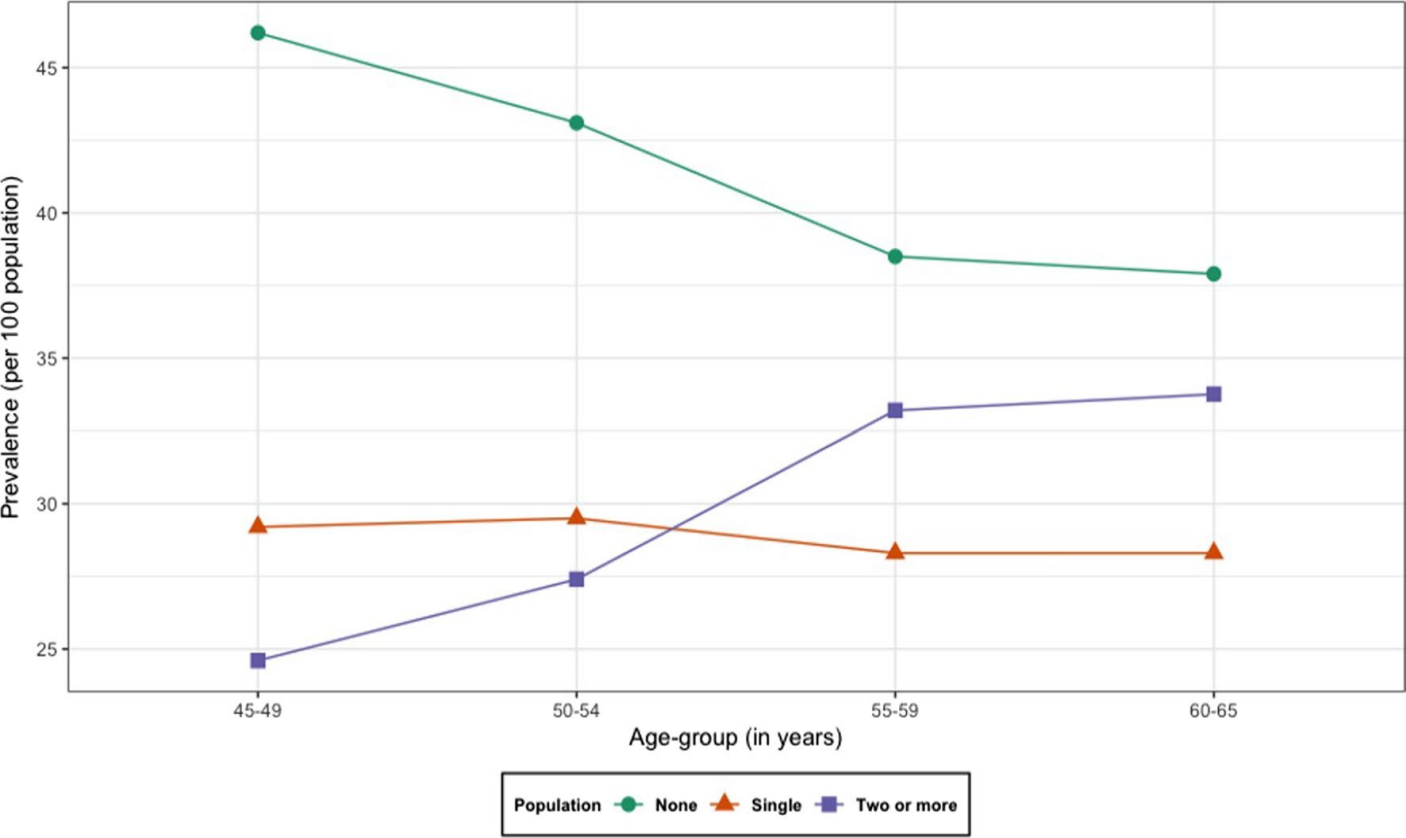
Prevalence of single and multimorbidity by age-group women in mid-life (45–65 years), Longitudinal Ageing Study in India (LASI), wave-1, 2017–18

Figure 3 suggests that prevalence of multimorbidity increases with age. The prevalence of multimorbidity for women aged 45–49, 50–54, 55–59 and 60–65 years was reported as 24.6, 27.4, 33.2 and 33.7 per 100 women. As women age, the cumulative number of chronic conditions too increase leading to an overall rise in multimorbidity prevalence. Thus, an influence of age is visible on the burden of multimorbidity among middle aged women in India.

### Variation in single and multimorbidity burden by states and union territories

**Fig. 4:**
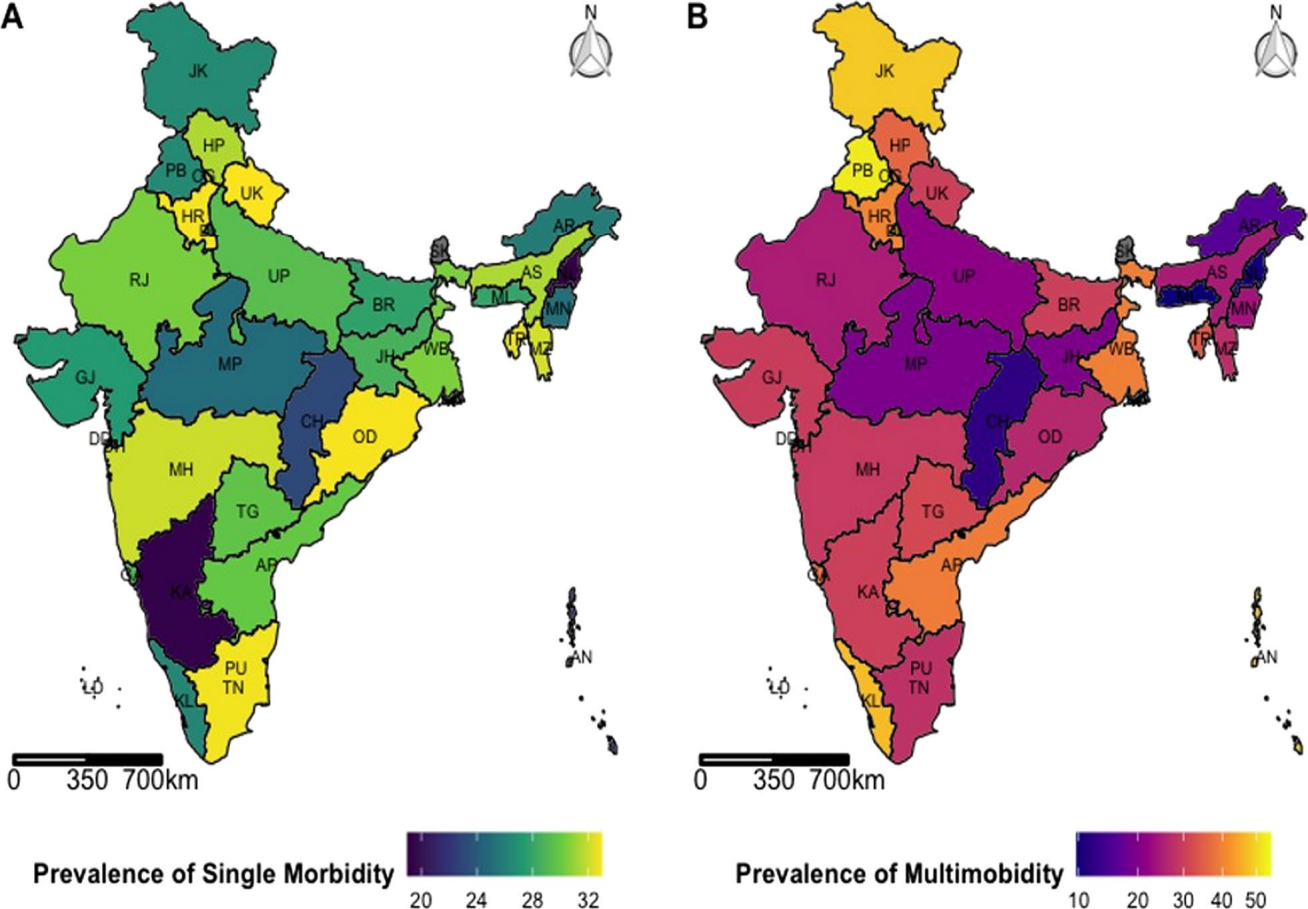
Prevalence of A. Single morbidity and B. Multimorbidity among women in mid-life (45–65 years) across different States/Union Territories, Longitudinal Ageing Study in India (LASI), wave-1, 2017–18

Figure 4A, B provides the age-standardised geographical distribution of single and multimorbidity burden over 35 states and union territories (except Sikkim, as data was not collected). Sixteen states/UTs reported the prevalence of single morbidity higher than the national average (Prevalence = 28.8 per 100 women). It is worth mentioning that four of these states belonged to the Northern region, followed by three states/UTs from North-eastern, Western and Southern region each. Two states/UTs belonged to the Eastern region, while one hailed form the central region of the country. The highest burden was reported by the states/UTs of Uttarakhand (Prevalence = 32.9 per 100 women), Odisha (Prevalence = 32.9 per 100 women), Haryana (Prevalence = 32.8 per 100 women), Tamil Nadu (Prevalence = 32.7 per 100 women) and Tripura (Prevalence = 32.6 per 100 women).

Nineteen states/UTs reported the prevalence of multimorbidity higher than the national average (Prevalence = 29.8 per 100 women). Notably, seven of these states/UTs belonged to the Northern and Southern region each, followed by two states/UTs from Eastern and Western region each. One state was from the North-eastern region of the country. The states/UTs of Chandigarh (Prevalence = 54.8 per 100 women), Punjab (Prevalence = 52.8 per 100 women), Andaman and Nicobar (Prevalence = 48.7 per 100 women), Jammu and Kashmir (Prevalence = 48.7 per 100 women) and Kerala (Prevalence = 47.2 per 100 women) reported highest prevalence of multimorbidity among middle aged women in India.

### Correlates of multimorbidity among midlife women in India

Table 3 illustrates the results from the bivariate analysis. The results suggest that age, women’s residence, religion, social group, level of education, occupation, wealth, consumption of tobacco and alcohol, physical activity, waist-hip ratio, marital status, family history of chronic diseases, and menopause experience were significantly associated with Chronic Disease Score.

**Table 3:** Bivariate analysis of chronic disease score among women in mid-life years (45–65 years), Longitudinal Ageing Study in India (LASI), wave-1, 2017–18

Correlates	Unadjusted odds ratio (UOR)	*p*-value	95% Confidence interval	
			Lower	Upper
Age (in years)	1.02	< 0.0001	1.01	1.04
*Residence*				
Rural (Ref.)	1.00			
Urban	1.83	< 0.0001	1.56	2.16
*Religion*				
Hindu (Ref.)	1.00			
Muslim	1.61	< 0.0001	1.39	1.85
Christian	0.81	0.488	0.42	1.49
Others	1.56	< 0.0001	1.29	1.87
*Social group*				
Scheduled castes (Ref.)	1.00			
Scheduled tribes	0.50	< 0.0001	0.43	0.58
Other backward class	1.05	0.422	0.42	0.92
Other castes	1.65	< 0.0001	1.47	1.86
*Level of education*				
No education (Ref.)	1.00			
Less than primary	1.61	< 0.0001	1.43	1.82
Primary completed	1.58	< 0.0001	1.37	1.82
Middle completed	1.81	< 0.0001	1.55	2.11
Matric completed	2.39	< 0.0001	1.93	2.96
Intermediate complete	0.81		0.33	1.95
Above intermediate	2.56	< 0.0001	1.7	3.87
*Occupation*				
Unemployed (Ref.)	1.00			
Blue collar	0.52	< 0.0001	0.47	0.58
White collar	0.98	0.951	0.57	1.69
Pink collar	0.78	0.055	0.6	1.01
Not classified/others	0.57	< 0.0001	0.49	0.67
*Wealth*				
Poor (Ref.)	1.00			
Middle	1.23	< 0.0001	1.06	1.42
Rich	1.75	< 0.0001	1.55	1.98
*Consumption of tobacco*				
Tobacco abstainer (Ref.)	1.00			
Only smoking	0.86	0.095	0.78	1.02
Only smokeless	0.89	0.099	0.79	1.02
Both smoke and smokeless tobacco	1.56	0.101	0.91	2.65
*Ever use alcohol*				
No (Ref.)				
Yes	0.49	< 0.0001	0.39	0.6
*Physical activity*				
Physically active (Ref.)	1.00			
Physically inactive	1.10	0.064	0.99	1.22
Waist-hip ratio	9.91	< 0.0001	5.66	15.56
*Marital status*				
Currently in union (Ref.)	1.00			
Not in union	0.97	0.697	0.85	1.11
Parity	0.96	< 0.05	0.94	0.99
*History of chronic disease*				
No (Ref.)	1.00			
Yes	2.30	< 0.0001	2.04	2.59
*Living arrangements*				
Living alone or with others (Ref.)	1.00			
Living with family members	1.21	0.229	0.88	1.65
*Experienced menopause*				
No (Ref.)	1.00			
Yes	1.06	0.352	0.93	1.21
Don't know	1.17	0.090	0.97	1.41

Dependent variable: Chronic Disease Score with three categories viz. No morbidity, Single Morbidity and Multimorbidity

Findings from multivariable analysis are presented in Table 4. These findings suggested age, place of residence, religion, social group, level of education, occupation, wealth, tobacco consumption, waist-hip ratio, history of chronic disease and experienced menopause in family were significantly associated with single morbidity among middle aged women in India.

**Table 4:** Multivariable analysis of chronic disease score among women in mid-life years (45–65 years), Longitudinal Ageing Study in India (LASI), wave-1, 2017–18

Correlates	Adjusted odds ratio (AOR)	*p*-value	95% Confidence interval	
			Lower	Upper
Age (in years)	1.02	< 0.0001	1.02	1.04
*Residence*				
Rural (Ref.)	1.00			
Urban	1.56	< 0.0001	1.39	1.76
*Religion*				
Hindu (Ref.)	1.00			
Muslim	1.29	< 0.0001	1.09	1.52
Christian	0.96	0.880	0.61	1.53
Others	0.88	0.329	0.69	1.13
*Social group*				
Scheduled castes (Ref.)	1.00			
Scheduled tribes	0.67	< 0.0001	0.55	0.81
Other backward class	0.96	0.643	0.85	1.10
Other castes	1.12	0.098	0.97	1.28
*Level of education*				
No education (Ref.)	1.00			
Less than primary	1.37	< 0.0001	1.21	1.57
Primary completed	1.19	< 0.0001	1.02	1.39
Middle completed	1.19	< 0.05	1.10	1.41
Matric completed	1.36	< 0.05	1.03	1.79
Intermediate complete	0.49	0.057	0.23	1.02
Above intermediate	1.39	0.128	0.90	2.13
*Occupation*				
Unemployed (Ref.)	1.00			
Blue collar	0.74	< 0.0001	0.66	0.83
White collar	0.81	0.466	0.48	1.04
Pink collar	0.71	< 0.0001	0.55	0.91
Not classified/others	0.73	< 0.0001	0.62	0.86
*Wealth*				
Poor (Ref.)	1.00			
Middle	1.13	0.052	0.99	1.28
Rich	1.56	< 0.0001	1.38	1.76
*Consumption of tobacco*				
Tobacco abstainer (Ref.)	1.00			
Only smoking	1.08	0.401	0.90	1.29
Only smokeless	1.08	0.238	0.95	1.22
Both smoke and smokeless tobacco	2.22	< 0.0001	1.27	3.90
*Ever use alcohol*				
No (Ref.)	1.00			
Yes	0.84	0.162	0.67	1.07
*Physical activity*				
Physically active (Ref.)	1.00			
Physically inactive	1.07	0.185	0.96	1.18
Waist-hip ratio	17.91	< 0.0001	9.86	32.51
*Marital status*				
Currently in union (Ref.)	1.00			
Not in union	1.01	0.859	0.89	1.14
Parity	1.01		0.99	1.03
*History of chronic disease*				
No (Ref.)	1.00			
Yes	2.02	< 0.0001	1.81	2.25
*Living arrangements*				
Living alone or with others (Ref.)	1.00			
Living with family members	1.19	1.66	0.96	1.46
*Experienced menopause*				
No (Ref.)	1.00			
Yes	1.24	< 0.0001	1.06	1.45
Don't know	1.37	< 0.0001	1.09	1.72

(1) Dependent variable: Chronic Disease Score with three categories viz. No morbidity, Single Morbidity and Multimorbidity

(2) All the estimates are adjusted for sub-national level (state) variation

For a one-year increase in age, the odds of multimorbidity versus the combined single and no morbidity were 1.02 times (OR 1.02, 95% CI 1.02–1.04) greater, given the other variables are held constant. Similarly, for urban residence, the odds of multimorbidity versus the combined single and no morbidity were 1.56 times (OR 1.56, CI 1.39–1.76) greater than rural residence, given the other variables are held constant in the model. For Scheduled Tribes, the odds of multimorbidity versus the combined single and no morbidity were 0.67 times (OR 0.67, CI 0.55–0.81) lower than Scheduled Castes, given the other variables were held constant in the model. For primary education completed, the odds of multimorbidity versus the combined single and no morbidity were 1.19 times (OR 1.19, CI 1.02–1.39) greater than women with no education, given the other variables were held constant in the model. For women in blue collar jobs, the odds of multimorbidity versus the combined single and no morbidity were 0.74 times (OR 0.74, CI 0.66–0.83) greater than women who were unemployed. Similarly, for women in pink collar jobs, the odds of multimorbidity versus the combined single and no morbidity were 0.71 times (OR 0.71, CI 0.55–0.91) greater than women who were unemployed, given the other variables were held constant in the model. For women belonging to affluent classes, the odds of multimorbidity versus the combined single and no morbidity were 1.56 times (OR 1.56, CI 138–1.76) greater than women who belonged to poor wealth background.

For a one unit increase in waist-hip ratio, the odds of multimorbidity versus the combined single and no morbidity were 17.91 times (OR 17.91, 95% CI 9.86–32.51) greater, given the other variables were held constant. For women with family history of chronic disease, the odds of multimorbidity versus the combined single and no morbidity were 2.02 times (OR 2.02, CI 1.81–2.25) greater than women who did not have the history, given the other variables were held constant in the model. For women who had experienced menopause, the odds of multimorbidity versus the combined single and no morbidity were 1.24 times (OR 1.24, CI 1.06–1.45) greater than women who did not experience menopause. Additional file 1: Tables S1, S2 and S3 provides the full model including the estimates showing sub-national level variation. This multivariate analysis was adjusted for state-level variation as the results from Fig. 4 depict sub-national variation in the burden of single and multimorbidity. In addition, we have presented two separate binary logistic regression models, (1) identifying the correlates of multimorbidity taking no multimorbidity as base (includes all mid-life women with no or single morbidity) and (2) identifying the correlates of any morbidity (including all mid-life women with single or multimorbidity) considering no morbidity as base.

### Multivariable associations between single and multimorbidity with indicators of health-related quality of life (HRQoL)

**Fig. 5:**
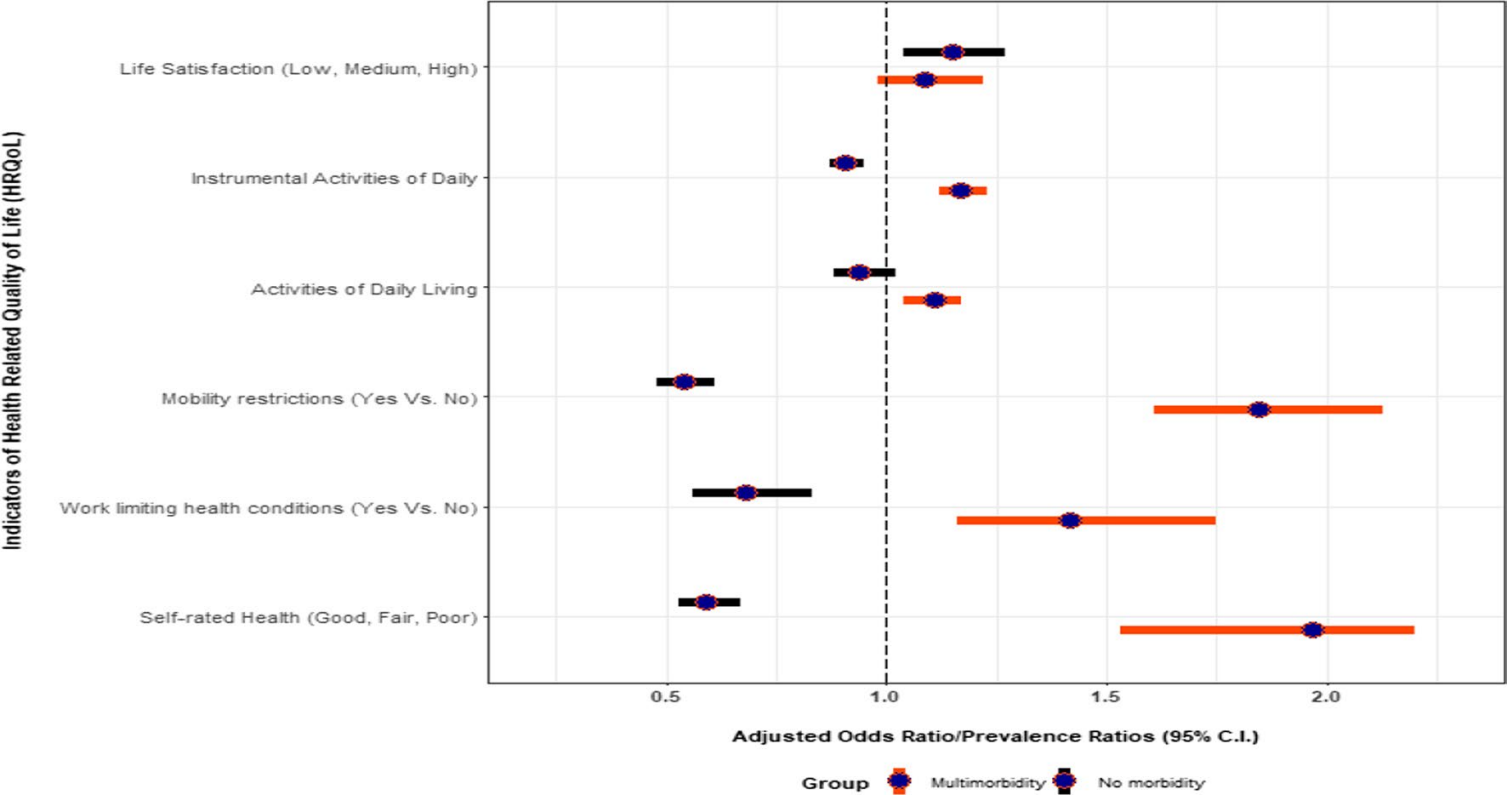
Association of single and multimorbidity with selected indicators of health-related quality of life (HRQoL), Longitudinal Ageing Study in India (LASI), wave-1, 2017–18

To study the linkages between multimorbidity and HRQoL, the present study included six indicators, the findings of which are highlighted in Fig. 5. The results suggest that five indicators were significantly associated with multimorbidity. For women who had no morbidity, the odds of reporting poor self-rated health versus the combined good and fair self-rated health were 0.59 times (OR 0.59, CI 0.53–0.67) lower than women who had single morbidity. However, for women who had multimorbidity, the odds of reporting poor self-rated health versus the combined good and fair self-rated health were 1.97 times (OR 1.97, CI 1.53- 2.20) higher than women who had single morbidity.

The likelihood of reporting at least one work limiting (OR 0.68, CI 0.56–0.83) health condition was lower for women with no morbidity as compared to women who reported single work limiting health condition. The likelihood of reporting at least one work limiting (OR 1.42, CI 1.16–1.75) health condition was greater for women with multimorbidity as compared to women who reported single morbidity.

The likelihood of reporting mobility restrictions (OR 0.54, C.I: 0.48–0.61) was lower for women with no morbidity as compared to women who reported single morbidity. The likelihood of reporting mobility restrictions (OR 1.85, CI 1.61–2.13) was higher for women with multimorbidity as compared to women who reported single morbidity.

The expected number of ADL is 1.11 times higher (PR 1.11, CI 1.04–1.17) for women with multimorbidity as compared to women who reported single morbidity, while holding all other variables in the model constant. The expected number of IADL is 0.91 times lower (PR 0.91, CI 0.87–0.95) for women with no morbidity as compared to women who reported single morbidity, while holding all other variables in the model constant. Similarly, the expected number of ADL was 1.17 times higher (PR 1.17, CI 1.12–1.23) for women with multimorbidity as compared to women who reported single morbidity, while holding all other variables in the model constant. Additional file 2: Tables S4, S5, S6, S7, S8 and S9 provides the results from full multivariable regression models.

## Discussion

The needs of women residing in LMICs such as India are often complex, attributed to the social and cultural disadvantages [26]. Hence, we undertook this study to estimate the burden of multimorbidity and its correlates among women in midlife using nationally representative data to provide comprehensive evidence. This study indicated hypertension (PR 27.1%) to be the most prevalent chronic condition, followed by gastrointestinal disorders (PR 17.9%) and musculoskeletal disorders (PR 16.9%) among middle-aged women. At the same time, Chandigarh (PR = 54.8 per 100 women) and Punjab (PR = 52.8 per 100 women) reported the highest prevalence of multimorbidity. This study provided a comprehensive overview of multimorbidity and its linkages with six indicators of HRQoL, five of which except (life satisfaction) were found to be significantly associated with multimorbidity. Here, it is worth noting, to the best of our knowledge this is the first study that highlighted multimorbidity among middle-aged women in India.

Our study indicated hypertension to be the most prevalent chronic condition, followed by gastrointestinal disorders and musculoskeletal disorders in contrast to the least prevalent cancer and chronic renal disease. These prevalent conditions are in harmony with the findings (skin diseases and hypertension were the most prevalent conditions) of our previous study, which intended to investigate multimorbidity among the working age-group in Odisha [27]. Prudently, this would require regular monitoring of health. Also, with the onset of NCDs, patients start navigating to the healthcare system. Here, this phase is a window of opportunity for giving health messages and motivating them to adopt healthy practices.

The prevalence of multimorbidity ranged from 20.79% to 31.27% among 45–50 and 60–65 years of age, respectively. This implies that chronic conditions start from an early age and accumulate with ageing. This is consistent with the findings of our previous study among women in the reproductive age group using data from the Demographic and Health Survey, 2015–16, which suggested an increase in multimorbidity from 0.5 per 100 women to 10.3 per 100 women across 15 to 49 years of age [21]. This manifests a need for early intervention as multimorbidity surfaces around reproductive years and cumulates to reach an alarming height during midlife surprising the individual. Current public health programs such as RMNCH + A for women’s health focus on sexual and reproductive needs with almost no attention garnered to post-menopausal health. Although, both the age groups should equally be targeted through expanded programs based on the needs and evidence.

Midlife marks a transition phase for women who face predisposed physiological and psychological changes around this period [28]. The healthcare needs of this group are complex and may require preventive, therapeutic, and emotional support, as evident in conditions such as depression [29]. The potential to which these changes affect an individual depends on various socio-demographic, cultural and environmental factors [30]. The present study identified various correlates associated with the number of chronic diseases such as being non-poor, consumption of tobacco, increased body mass index, waist-hip ratio, age at marriage, marital status, parity and history of chronic disease. These findings were homogenous with the observations of a similar study conducted amongst middle-aged women in Brazil, an LMIC with similar demography to India [31]. Here, it should be taken into cognizance that the lack of literature on multimorbidity among middle-aged women in India made comparing our findings with similar studies challenging.

Further, we explored regional variations in prevalence of multimorbidity which elucidates nineteen states/UTs have a higher prevalence of multimorbidity than the national average (PR 29.8 per 100 women), with states like Punjab and Kerala reporting the highest prevalence. Both Punjab and Kerala are among the most developed states in terms of per capita income, which also complements our finding that multimorbidity prevails among non-poor sections. In India, health is a state subject with disproportionate health infrastructure and availability across states. These state-level differences can also be attributed to the underlying social and cultural barriers such as non-prioritizing women’s health [32]. Since, women’s needs are often overshadowed, it is imperative to make them aware of their health to address the barriers to self-care and prioritization. Primary healthcare facilities need to be strengthened to improve coordinated quality care availability, accessibility and affordability (3A).

Previous evidence suggests multimorbidity is associated with inferior HRQoL among women than men at midlife; which could be attributed to menopausal symptoms as well as onset of chronic conditions simultaneously at this age [13]. This could lead to a higher multimorbidity among females than their age matched counterparts. Also, gender differences may affect adapting to the changes in midlife leading to poorer HRQoL among women. Five indicators of HRQoL such as poor self-rated health [RRR 2.56 (2.15, 3.04)], work-limiting health conditions [OR 1.90 (1.53, 2.36)], mobility [3.31(2.87, 3.80)], activities of daily living [OR 2.31 (1.92, 2.79)], instrumental activities of daily living [1.73 (1.53, 1.96)] were found to be significantly associated with multimorbidity. These findings are in harmony with a similar study on quality of life among midlife women in the United States which reported QoL decline significantly with each added condition [32]. Deteriorated HRQoL can be attributed to additional stress among women as they need to care for the dependents at home and inaccessibility of health services and various other gender norms [33].

### Strengths and limitations

The estimates provided in this study are based on nationally representative data from LASI, 2017–18. The study’s major strength is that it employed a list of seventeen NCDs to generate empirical evidence on single and multimorbidity for middle-aged women in India. Furthermore, the study highlighted patterns and sub-national level variation in the burden of single and multimorbidity. The study also examines the linkages between single and multimorbidity with selected indicators of HRQoL. However, the study is based on self-reported data, which might have caused misclassification bias in the study estimates. Also, as the study employs only one round of a longitudinal survey, it does not capture causality.

### Implications of the findings

Previous studies suggest adopting a healthy lifestyle in midlife has substantial overall health benefits [34]. The healthcare needs for countering non-communicable disease multimorbidity are usually unmet among women. The shared risk factors identified by this study need to be targeted and reduced. Health promotion through behavioural change communication for abstaining from tobacco, alcohol and physical activities should be encouraged. Emphasis should be laid on screening by expanding current programs such as the National Programme for Prevention and Control of Cancer, Diabetes, Cardiovascular Diseases, and Stroke (NPCDCS). A holistic care approach be developed for women’s overall well-being, which would encompass true universal health coverage. This can be achieved by integrating current programs such as NPCDCS and RMNCH + A and expanding it for midlife women. Health and Wellness Centres provides a comprehensive range of services easily available at a nearby facility envisaged under one umbrella. Women should be made aware of countering age stereotypes and motivated for healthy living. Monthly house-to-house visits by frontline workers such as ASHA can help us know the well-being of women. Also, there is a need to involve women of this age group in the family to feel worthy. These women are often not aware of services or cannot avail may be due the lack of digital literacy. Hence, safe transportation for accessing healthcare facilities should be readily available. Self-help women groups can be roped in as a platform for community engagement and delivering health messages.

## Conclusion

This study suggests multimorbidity is increasingly prevalent in midlife women associated with inferior quality of life, which cannot be overlooked. These findings suggest an integration between NPCDCS and RMNCH + A by expanding its horizon for midlife women, thus focusing on a life course approach. Future studies are required to explore the linkages in this age group.

## Supplementary Information

**Additional file 1**. Multivariable analysis of chronic disease score among women in mid-life years (45–65 years).

**Additional file 2**. Multivariable associations between single and multimorbidity with indicators of health-related quality of life (HRQoL).

## Data Availability

The dataset analysed during the current study is available in the LASI data repository held at ICT, IIPS [https://iipsindia.ac.in/content/lasi-wave-i]. Requests to access the data should be made to datacenter@ipsindia.ac.in.

## Abbreviations

ADL: Activities of daily living
CDS: Chronic disease score
HRQoL: Health related quality of life
IADL: Instrumental activities of daily living
LASI: Longitudinal ageing study in India
LMICs: Low-and-middle-income countries
NCDs: Non-communicable diseases
NHM: National Health Mission
RMNCH + A: Reproductive, maternal, neonatal, child and adolescent health
SRH: Self-rated health
WHO: World Health Organization
