# Supplementary material for: Multimorbidity among midlife women in India: well-being beyond reproductive age
Source: BMC Womens Health. 2022 Apr 12;22:117. doi: 10.1186/s12905-022-01693-2 (PMC9004080; doi:10.1186/s12905-022-01693-2)
Supplement: Supplementary file 1 — Additional file 1. Multivariable analysis of chronic disease score among women in mid-life years (45–65 years). [file 12905_2022_1693_MOESM1_ESM.docx]

**Supplementary Tables**

| **Supplementary Table S1.** Multivariable analysis of chronic disease score among women in mid-life years (45-65 years), Longitudinal Ageing Study in India (LASI), wave-1, 2017-18. | | | | |
| --- | --- | --- | --- | --- |
| **Correlates** | **Adjusted Odds Ratio (AOR)** | **p-value** | **95% Confidence Interval** | |
|  |  |  | **Lower** | **Upper** |
| **Age (in years)** | 1.02 | p<0.0001 | 1.02 | 1.04 |
| **Residence** |  |  |  |  |
| Rural (Ref.) | 1.00 |  |  |  |
| Urban | 1.56 | p<0.0001 | 1.39 | 1.76 |
| **Religion** |  |  |  |  |
| Hindu (Ref.) | 1.00 |  |  |  |
| Muslim | 1.29 | p<0.0001 | 1.09 | 1.52 |
| Christian | 0.96 | p=0.880 | 0.61 | 1.53 |
| Others | 0.88 | p=0.329 | 0.69 | 1.13 |
| **Social Group** |  |  |  |  |
| Scheduled Castes (Ref.) | 1.00 |  |  |  |
| Scheduled Tribes | 0.67 | p<0.0001 | 0.55 | 0.81 |
| Other Backward Class | 0.96 | p=0.643 | 0.85 | 1.10 |
| Other Castes | 1.12 | p=0.098 | 0.97 | 1.28 |
| **Level of education** |  |  |  |  |
| No Education (Ref.) | 1.00 |  |  |  |
| Less than Primary | 1.37 | p<0.0001 | 1.21 | 1.57 |
| Primary Completed | 1.19 | p<0.0001 | 1.02 | 1.39 |
| Middle Completed | 1.19 | p<0.05 | 1.10 | 1.41 |
| Matric Completed | 1.36 | p<0.05 | 1.03 | 1.79 |
| Intermediate Complete | 0.49 | p=0.057 | 0.23 | 1.02 |
| Above Intermediate | 1.39 | p=0.128 | 0.90 | 2.13 |
| **Occupation** |  |  |  |  |
| Unemployed (Ref.) | 1.00 |  |  |  |
| Blue Collar | 0.74 | p<0.0001 | 0.66 | 0.83 |
| White Collar | 0.81 | p=0.466 | 0.48 | 1.04 |
| Pink Collar | 0.71 | p<0.0001 | 0.55 | 0.91 |
| Not Classified/Others | 0.73 | p<0.0001 | 0.62 | 0.86 |
| **Wealth** |  |  |  |  |
| Poor (Ref.) | 1.00 |  |  |  |
| Middle | 1.13 | p=0.052 | 0.99 | 1.28 |
| Rich | 1.56 | p<0.0001 | 1.38 | 1.76 |
| **Consumption of tobacco** |  |  |  |  |
| Tobacco Abstainer (Ref.) | 1.00 |  |  |  |
| Only Smoking | 1.08 | p=0.401 | 0.90 | 1.29 |
| Only Smokeless | 1.08 | p=0.238 | 0.95 | 1.22 |
| Both smoke and smokeless tobacco | 2.22 | p<0.0001 | 1.27 | 3.90 |
| **Ever use alcohol** |  |  |  |  |
| No (Ref.) | 1.00 |  |  |  |
| Yes | 0.84 | p=0.162 | 0.67 | 1.07 |
| **Physical Activity** |  |  |  |  |
| Physically active (Ref.) | 1.00 |  |  |  |
| Physically inactive | 1.07 | p=0.185 | 0.96 | 1.18 |
| **Waist-Hip Ratio** | 17.91 | p<0.0001 | 9.86 | 32.51 |
| **Marital Status** |  |  |  |  |
| Currently in Union (Ref.) | 1.00 |  |  |  |
| Not in Union | 1.01 | p=0.859 | 0.89 | 1.14 |
| **Parity** | 1.01 |  | 0.99 | 1.03 |
| **History of Chronic Disease** |  |  |  |  |
| No (Ref.) | 1.00 |  |  |  |
| Yes | 2.02 | p<0.0001 | 1.81 | 2.25 |
| **Living Arrangements** |  |  |  |  |
| Living alone or with others (Ref.) | 1.00 |  |  |  |
| Living with family members | 1.19 | p=1.66 | 0.96 | 1.46 |
| **Experienced Menopause** |  |  |  |  |
| No (Ref.) | 1.00 |  |  |  |
| Yes | 1.24 | p<0.0001 | 1.06 | 1.45 |
| Don't Know | 1.37 | p<0.0001 | 1.09 | 1.72 |
| **State** |  |  |  |  |
| Jammu and Kashmir (Ref.) | 1.00 |  |  |  |
| Himachal Pradesh | 1.12 | p=0.488 | 0.81 | 1.53 |
| Punjab | 1.87 | p<0.0001 | 1.32 | 2.63 |
| Chandigarh | 1.14 | p=0.471 | 0.79 | 1.66 |
| Uttarakhand | 1.02 | p=0.896 | 0.75 | 1.38 |
| Haryana | 1.20 | p<0.05 | 1.07 | 1.90 |
| Delhi | 1.16 | p=0.410 | 0.81 | 1.65 |
| Rajasthan | 0.86 | p=0.321 | 0.65 | 1.14 |
| Uttar Pradesh | 0.67 | p<0.05 | 0.51 | 0.88 |
| Bihar | 0.93 | p=0.648 | 0.71 | 1.23 |
| Arunachal Pradesh | 0.84 | p=0.481 | 0.52 | 0.88 |
| Nagaland | 0.37 | p<0.0001 | 0.18 | 0.77 |
| Mizoram | 0.43 | p=0.652 | 0.63 | 2.05 |
| Tripura | 1.14 | p<0.05 | 1.02 | 1.96 |
| Meghalaya | 1.41 | p<0.05 | 0.34 | 0.92 |
| Assam | 0.56 | p<0.05 | 0.52 | 0.93 |
| West Bengal | 0.70 | p=0.248 | 0.89 | 1.53 |
| Jharkhand | 1.17 | p<0.05 | 0.57 | 0.98 |
| Odisha | 0.75 | p=0.247 | 0.89 | 1.57 |
| Chhattisgarh | 1.18 | p<0.0001 | 0.39 | 0.72 |
| Madhya Pradesh | 0.61 | p<0.0001 | 0.44 | 0.84 |
| Gujarat | 0.83 | p=0.237 | 0.62 | 1.12 |
| Daman and Diu | 1.02 | p=0.922 | 0.71 | 1.46 |
| Dadra and Nagar Haveli | 0.76 | p=0.143 | 0.53 | 1.09 |
| Maharashtra | 0.98 | p=0.942 | 0.74 | 1.31 |
| Andhra Pradesh | 1.80 | p<0.0001 | 1.34 | 2.43 |
| Karnataka | 0.66 | p=0.086 | 0.41 | 1.60 |
| Goa | 0.78 | p=0.141 | 0.56 | 1.08 |
| Lakshadweep | 0.76 | p=0.184 | 0.50 | 1.14 |
| Kerala | 0.82 | p=0.280 | 0.58 | 1.17 |
| Tamil Nadu | 1.01 | p=0.915 | 0.75 | 1.37 |
| Puducherry | 1.29 | p=0.149 | 0.91 | 1.83 |
| Andaman and Nicobar Islands | 0.06 | p<0.0001 | 1.51 | 3.70 |
| Telangana | 1.35 | p<0.05 | 1.02 | 1.81 |

| **Supplementary Table S2**. Binary logistic regression analysis of multimorbidity (Multimorbidity Vs. No multimorbidity) among women in mid-life years (45-65 years), Longitudinal Ageing Study in India (LASI), wave-1, 2017-18 | | | | | |
| --- | --- | --- | --- | --- | --- |
| **Correlates** | **Adjusted Odds Ratio (AOR)** | **p-value** |  | **95% Confidence Interval** | |
|  |  |  |  | **Lower** | **Upper** |
| **Age (in years)** | 1.03 | p<0.0001 |  | 1.02 | 1.04 |
| **Residence** |  |  |  |  |  |
| Rural (Ref.) | 1.00 |  |  |  |  |
| Urban | 1.59 | p<0.0001 |  | 1.38 | 1.82 |
| **Religion** |  |  |  |  |  |
| Hindu (Ref.) | 1.00 |  |  |  |  |
| Muslim | 1.25 | p=0.020 |  | 1.04 | 1.52 |
| Christian | 1.04 | p=0.828 |  | 0.69 | 1.58 |
| Others | 0.85 | p=0.237 |  | 0.65 | 1.11 |
| **Social Group** |  |  |  |  |  |
| Scheduled Castes (Ref.) | 1.00 |  |  |  |  |
| Scheduled Tribes | 0.67 | p<0.0001 |  | 0.53 | 0.85 |
| Other Backward Class | 1.08 | p<0.0001 |  | 0.91 | 1.28 |
| Other Castes | 1.24 | p<0.0001 |  | 1.06 | 1.46 |
| **Level of education** |  |  |  |  |  |
| No Education (Ref.) | 1.00 |  |  |  |  |
| Less than Primary | 1.33 | p<0.0001 |  | 1.13 | 1.56 |
| Primary Completed | 1.23 | p=0.021 |  | 1.03 | 1.45 |
| Middle Completed | 1.19 | p=0.067 |  | 0.98 | 1.45 |
| Matric Completed | 131 | p=0.102 |  | 0.94 | 1.8 |
| Intermediate Complete | 0.64 | p=0.164 |  | 0.34 | 1.19 |
| Above Intermediate | 1.52 | p=0.054 |  | 0.99 | 2.34 |
| **Occupation** |  |  |  |  |  |
| Unemployed (Ref.) | 1.00 |  |  |  |  |
| Blue Collar | 0.68 | p<0.0001 |  | 0.59 | 0.78 |
| White Collar | 0.71 | p=0.191 |  | 0.42 | 1.19 |
| Pink Collar | 0.7 | p=0.045 |  | 0.5 | 0.99 |
| Not Classified/Others | 0.68 | p<0.0001 |  | 0.56 | 0.83 |
| **Wealth** |  |  |  |  |  |
| Poor (Ref.) | 1.00 |  |  |  |  |
| Middle | 1.09 | p=0.230 |  | 0.94 | 1.26 |
| Rich | 1.59 | p<0.0001 |  | 1.38 | 1.83 |
| **Consumption of tobacco** |  |  |  |  |  |
| Tobacco Abstainer (Ref.) | 1.00 |  |  |  |  |
| Only Smoking | 0.93 | p=0.558 |  | 0.73 | 1.18 |
| Only Smokeless | 1.12 | p=0.121 |  | 0.96 | 1.31 |
| Both smoke and smokeless tobacco | 2.43 | p<0.0001 |  | 1.37 | 4.3 |
| **Ever use alcohol** |  |  |  |  |  |
| No (Ref.) | 1.00 |  |  |  |  |
| Yes | 0.90 | p=0.479 |  | 0.67 | 1.2 |
| **Physical Activity** |  |  |  |  |  |
| Physically active (Ref.) | 1.00 |  |  |  |  |
| Physically inactive | 1.12 | p=0.055 |  | 0.99 | 1.25 |
| **Waist-Hip Ratio** | 32.73 | p<0.0001 |  | 16.08 | 66.61 |
| **Marital Status** |  |  |  |  |  |
| Currently in Union (Ref.) | 1.00 |  |  |  |  |
| Not in Union | 0.98 | p=0.830 |  | 0.85 | 1.14 |
| **Parity** | 1.02 | p=0.136 |  | 0.99 | 1.04 |
| **History of Chronic Disease** |  |  |  |  |  |
| No (Ref.) | 1.00 |  |  |  |  |
| Yes | 2.01 | p<0.0001 |  | 1.78 | 2.27 |
| **Living Arrangements** |  |  |  |  |  |
| Living alone or with others (Ref.) | 1.00 |  |  |  |  |
| Living with family members | 1.23 | p=0.079 |  | 0.97 | 1.54 |
| **Experienced Menopause** |  |  |  |  |  |
| No (Ref.) | 1.00 |  |  |  |  |
| Yes | 1.26 | p<0.0001 |  | 1.04 | 1.53 |
| Don't Know | 1.32 | p=0.046 |  | 1.01 | 1.73 |
| **State** |  |  |  |  |  |
| Jammu and Kashmir (Ref.) | 1.00 |  |  |  |  |
| Himachal Pradesh | 1.13 | p=0.519 |  | 0.78 | 1.62 |
| Punjab | 1.92 | p<0.0001 |  | 1.31 | 2.8 |
| Chandigarh | 1.21 | p=0.352 |  | 0.81 | 1.78 |
| Uttarakhand | 0.94 | p=0.762 |  | 0.66 | 1.34 |
| Haryana | 1.20 | p=0.057 |  | 0.99 | 1.91 |
| Delhi | 1.14 | p=0.504 |  | 0.77 | 1.69 |
| Rajasthan | 0.83 | 0.295 |  | 0.59 | 1.17 |
| Uttar Pradesh | 0.64 | p<0.0001 |  | 0.47 | 0.87 |
| Bihar | 1.02 | p=0.864 |  | 0.75 | 1.4 |
| Arunachal Pradesh | 0.8 | p=0.455 |  | 0.44 | 1.43 |
| Nagaland | 0.34 | p=0.041 |  | 0.12 | 0.95 |
| Manipur | 0.44 | p=0.001 |  | 0.27 | 0.71 |
| Mizoram | 1.07 | p=0.815 |  | 0.58 | 1.99 |
| Tripura | 1.34 | p=0.130 |  | 0.92 | 1.96 |
| Meghalaya | 0.36 | p<0.0001 |  | 0.19 | 0.66 |
| Assam | 0.64 | p=0.012 |  | 0.46 | 0.91 |
| West Bengal | 1.19 | p=0.277 |  | 0.87 | 1.61 |
| Jharkhand | 0.71 | p=0.041 |  | 0.51 | 0.98 |
| Odisha | 1.04 | p=0.818 |  | 0.74 | 1.45 |
| Chhattisgarh | 0.49 | p<0.0001 |  | 0.34 | 0.71 |
| Madhya Pradesh | 0.64 | p=0.017 |  | 0.43 | 0.92 |
| Gujarat | 0.87 | p=0.452 |  | 0.62 | 1.22 |
| Daman and Diu | 1.08 | p=0.708 |  | 0.71 | 1.64 |
| Dadra and Nagar Haveli | 0.68 | p=0.111 |  | 0.43 | 1.9 |
| Maharashtra | 0.94 | p=0.726 |  | 0.68 | 1.3 |
| Andhra Pradesh | 0.89 | p<0.0001 |  | 1.36 | 2.63 |
| Karnataka | 0.84 | p=0.517 |  | 0.51 | 1.42 |
| Goa | 0.77 | p=0.163 |  | 0.53 | 1.11 |
| Lakshadweep | 0.99 | p=0.977 |  | 0.64 | 1.55 |
| Kerala | 0.81 | p=0.238 |  | 0.55 | 1.16 |
| Tamil Nadu | 0.96 | p=0.800 |  | 0.67 | 1.35 |
| Puducherry | 1.36 | p=0.118 |  | 0.92 | 2.01 |
| Andaman and Nicobar Islands | 2.55 | p<0.0001 |  | 1.63 | 4.01 |
| Telangana | 1.45 | p=0.027 |  | 1.04 | 2.02 |

| **Supplementary Table S3**. Binary logistic regression analysis of any morbidity (Any morbidity Vs. No morbidity) among women in mid-life years (45-65 years), Longitudinal Ageing Study in India (LASI), wave-1, 2017-18 | | | | |
| --- | --- | --- | --- | --- |
| **Correlates** | **Adjusted Odds Ratio (AOR)** | **p-value** | **95% Confidence Interval** | |
|  |  |  | **Lower** | **Upper** |
| **Age (in years)** | 1.02 | p<0.0001 | 1.01 | 1.03 |
| **Residence** |  |  |  |  |
| Rural (Ref.) | 1.00 |  |  |  |
| Urban | 1.55 | p<0.0001 | 1.36 | 1.78 |
| **Religion** |  |  |  |  |
| Hindu (Ref.) | 1.00 |  |  |  |
| Muslim | 1.34 | p<0.0001 | 1.11 | 1.62 |
| Christian | 0.94 | 0.819 | 0.58 | 1.53 |
| Others | 0.88 | 0.419 | 0.65 | 1.20 |
| **Social Group** |  |  |  |  |
| Scheduled Castes (Ref.) | 1.00 |  |  |  |
| Scheduled Tribes | 0.65 | p<0.0001 | 0.53 | 0.80 |
| Other Backward Class | 0.91 | 0.153 | 0.79 | 1.04 |
| Other Castes | 1.04 | 0.608 | 0.89 | 1.21 |
| **Level of education** |  |  |  |  |
| No Education (Ref.) | 1.00 |  |  |  |
| Less than Primary | 1.41 | p<0.0001 | 1.21 | 1.65 |
| Primary Completed | 1.15 | p=0.114 | 0.97 | 1.37 |
| Middle Completed | 1.20 | p=0.076 | 0.98 | 1.47 |
| Matric Completed | 1.44 | p=0.009 | 1.10 | 1.91 |
| Intermediate Complete | 0.46 | p=0.032 | 0.22 | 0.93 |
| Above Intermediate | 1.23 | p=0.329 | 0.81 | 1.85 |
| **Occupation** |  |  |  |  |
| Unemployed (Ref.) | 1.00 |  |  |  |
| Blue Collar | 0.76 | p<0.0001 | 0.67 | 0.86 |
| White Collar | 0.99 | p=0.986 | 0.53 | 1.88 |
| Pink Collar | 0.70 | p=0.025 | 0.52 | 0.96 |
| Not Classified/Others | 0.74 | p<0.0001 | 0.62 | 0.89 |
| **Wealth** |  |  |  |  |
| Poor (Ref.) | 1.00 |  |  |  |
| Middle | 1.19 | p=0.018 | 1.03 | 1.37 |
| Rich | 1.57 | p<0.0001 | 1.38 | 1.79 |
| **Consumption of tobacco** |  |  |  |  |
| Tobacco Abstainer (Ref.) | 1.00 |  |  |  |
| Only Smoking | 1.17 | p=0.155 | 0.94 | 1.45 |
| Only Smokeless | 1.06 | p=0.446 | 0.92 | 1.22 |
| Both smoke and smokeless tobacco | 1.96 | p=0.046 | 1.01 | 3.79 |
| **Ever use alcohol** |  |  |  |  |
| No (Ref.) | 1.00 |  |  |  |
| Yes | 0.81 | p=0.104 | 0.63 | 1.04 |
| **Physical Activity** |  |  |  |  |
| Physically active (Ref.) | 1.00 |  |  |  |
| Physically inactive | 1.04 | p=0.535 | 0.93 | 1.15 |
| **Waist-Hip Ratio** | 12.36 | p<0.0001 | 6.41 | 23.82 |
| **Marital Status** |  |  |  |  |
| Currently in Union (Ref.) | 1.00 |  |  |  |
| Not in Union | 1.05 | p=0.522 | 0.91 | 1.20 |
| **Parity** | 1.01 | p=0.604 | 0.98 | 1.03 |
| **History of Chronic Disease** |  |  |  |  |
| No (Ref.) | 1.00 |  |  |  |
| Yes | 2.05 | p<0.0001 | 1.82 | 2.30 |
| **Living Arrangements** |  |  |  |  |
| Living alone or with others (Ref.) | 1.00 |  |  |  |
| Living with family members | 1.17 | p=0.178 | 0.93 | 1.49 |
| **Experienced Menopause** |  |  |  |  |
| No (Ref.) | 1.00 |  |  |  |
| Yes | 1.26 | p=0.013 | 1.05 | 1.51 |
| Don't Know | 1.47 | p<0.0001 | 1.13 | 1.91 |
| **State** |  |  |  |  |
| Jammu and Kashmir (Ref.) | 1.19 | p=0.359 | 0.82 | 1.73 |
| Himachal Pradesh | 1.95 | p<0.0001 | 1.27 | 2.99 |
| Punjab | 1.06 | p=0.806 | 0.68 | 1.64 |
| Chandigarh | 1.13 | p=0.519 | 0.78 | 1.63 |
| Uttarakhand | 1.66 | p<0.0001 | 1.17 | 2.36 |
| Haryana | 1.19 | p=0.406 | 0.79 | 1.80 |
| Delhi | 0.93 | p=0.664 | 0.66 | 1.30 |
| Rajasthan | 0.74 | p=0.057 | 0.54 | 1.01 |
| Uttar Pradesh | 0.95 | p=0.771 | 0.69 | 1.32 |
| Bihar | 0.88 | p=0.629 | 0.51 | 1.50 |
| Arunachal Pradesh | 0.38 | p=0.012 | 0.17 | 0.81 |
| Nagaland | 0.45 | p<0.0001 | 0.29 | 0.70 |
| Mizoram | 1.25 | p=0.5 | 0.66 | 2.37 |
| Tripura | 1.55 | p=0.029 | 1.05 | 2.31 |
| Meghalaya | 0.66 | p=0.14 | 0.38 | 1.15 |
| Assam | 0.80 | p=0.19 | 0.57 | 1.12 |
| West Bengal | 1.25 | p=0.194 | 0.89 | 1.73 |
| Jharkhand | 0.84 | p=0.299 | 0.61 | 1.17 |
| Odisha | 1.36 | p=0.079 | 0.97 | 1.92 |
| Chhattisgarh | 0.57 | p<0.0001 | 0.40 | 0.80 |
| Madhya Pradesh | 0.64 | p=0.02 | 0.44 | 0.93 |
| Gujarat | 0.84 | p=0.318 | 0.59 | 1.18 |
| Daman and Diu | 1.09 | p=0.69 | 0.72 | 1.64 |
| Dadra and Nagar Haveli | 0.88 | p=0.529 | 0.58 | 1.32 |
| Maharashtra | 1.08 | p=0.662 | 0.77 | 1.50 |
| Andhra Pradesh | 1.85 | p=0.001 | 1.30 | 2.64 |
| Karnataka | 0.58 | p=0.023 | 0.36 | 0.93 |
| Goa | 0.82 | p=0.31 | 0.56 | 1.21 |
| Lakshadweep | 0.64 | p=0.047 | 0.41 | 0.99 |
| Kerala | 0.81 | p=0.306 | 0.53 | 1.22 |
| Tamil Nadu | 1.13 | p=0.487 | 0.80 | 1.61 |
| Puducherry | 1.34 | p=0.166 | 0.89 | 2.01 |
| Andaman and Nicobar Islands | 1.99 | p=0.007 | 1.20 | 3.29 |
| Telangana | 1.39 | p=0.06 | 0.99 | 1.95 |
