# Supplementary material for: Multimorbidity among midlife women in India: well-being beyond reproductive age
Source: BMC Womens Health. 2022 Apr 12;22:117. doi: 10.1186/s12905-022-01693-2 (PMC9004080; doi:10.1186/s12905-022-01693-2)
Supplement: Supplementary file 2 — Additional file 2. Multivariable associations between single and multimorbidity with indicators of health-related quality of life (HRQoL). [file 12905_2022_1693_MOESM2_ESM.docx]

**Supplementary Tables**

| **Supplementary Table S4.** Ordered logistic regression analysis of Self-rated Health (N=23,951) among women in mid-life years (45-65 years), Longitudinal Ageing Study in India (LASI), wave-1, 2017-18. | |
| --- | --- |
| **Correlates** | **Self-rated Health (Good, Fair, Poor)** |
| **Chronic Disease Score** |  |
| One (Single Disease) (Ref.) | 1.00 |
| Zero (None) | 0.59***(0.53, 0.67) |
| Two or more (Multimorbidity) | 1.97***(1.53, 2.20) |
| **Age (in years)** | 1.02***(1.01, 1.03) |
| **Residence** |  |
| Rural | 1.00 |
| Urban | 0.74***(0.66, 0.83) |
| **Religion** |  |
| Hindu | 1.00 |
| Muslim | 1.16 (0.97, 1.39) |
| Christian | 1.45 (0.9, 2.25) |
| Others | 0.99 (0.77, 1.27) |
| **Social Group** |  |
| Scheduled Castes | 1.00 |
| Scheduled Tribes | 0.93 (0.77, 1.13) |
| Other Backward Class | 0.92 (0.81, 1.03) |
| Other Castes | 0.86*(0.76, 0.98) |
| **Level of education** |  |
| No Education | 1.00 |
| Less than Primary | 1.07 (0.92, 1.24) |
| Primary Completed | 0.77***(0.67, 0.88) |
| Middle Completed | 0.79***(0.67, 0.94) |
| Matric Completed | 0.68***(0.53, 0.88) |
| Intermediate Complete | 1.58 (0.82, 3.04) |
| Above Intermediate | 0.36***(0.25, 0.53) |
| **Occupation** |  |
| Unemployed | 1.00 |
| Blue Collar | 0.92 (0.82, 1.02) |
| White Collar | 0.88(0.59, 1.30) |
| Pink Collar | 0.95 (0.71, 1.27) |
| Not Classified/Others | 1.19 (0.95 1.31) |
| **Wealth** |  |
| Poor | 1.00 |
| Middle | 0.93 (0.82, 1.06) |
| Rich | 1.00 (0.95, 1.31) |
| **Consumption of tobacco** |  |
| Tobacco Abstainer | 1.00 |
| Only Smoking | 1.48***(1.20, 1.83) |
| Only Smokeless | 0.97 (0.85, 1.12) |
| Both smoke and smokeless tobacco | 1.09 (0.57, 2.10) |
| **Ever use alcohol** |  |
| No | 1.00 |
| Yes | 1.07 (0.84, 1.37) |
| **Physical Activity** |  |
| Physically active | 1.00 |
| Physically inactive | 1.21***(1.10, 1.34) |
| **Waist-Hip Ratio** | 0.66 (0.36, 1.21) |
| **Marital Status** |  |
| Currently in Union | 1.00 |
| Not in Union | 1.17***(1.05, 1.32) |
| **Parity** | 1.01 (0.99, 1.04) |
| **History of Chronic Disease** |  |
| No (Ref.) | 1.00 |
| Yes | 1.19*** (1.07, 1.32) |
| **Living Arrangements** |  |
| Living alone or with others (Ref.) | 1.00 |
| Living with family members | 0.78** (0.63, 0.95) |
| **Experienced Menopause** |  |
| No (Ref.) | 1.00 |
| Yes | 1.18* (1.01, 1.37) |
| Don't Know | 1.38***(1.09, 1.75) |
| **State** |  |
| Jammu and Kashmir (Ref.) | 1.00 |
| Himachal Pradesh | 1.15 (0.80, 1.63) |
| Punjab | 1.61*** (1.12, 2.30) |
| Chandigarh | 0.72 (0.49, 1.06) |
| Uttarakhand | 1.20 )0.87, 1.64) |
| Haryana | 0.73 (0.49, 1.06) |
| Delhi | 0.53***(0.35, 0.78) |
| Rajasthan | 0.58***(0.42, 0.81) |
| Uttar Pradesh | 1.16 (0.86, 1.58) |
| Bihar | 0.72*(0.53, 0.97) |
| Arunachal Pradesh | 0.26***(0.15, 0.45) |
| Nagaland | 0.35***(0.17, 0.73) |
| Mizoram | 0.42***(0.23, 0.76) |
| Tripura | 1.41*(1.01, 1.99) |
| Meghalaya | 0.16***(0.09, 0.27) |
| Assam | 0.93 (0.67, 1.27) |
| West Bengal | 1.77***(1.2, 2.38) |
| Jharkhand | 0.67*(0.49, 0.93) |
| Odisha | 0.93 (0.67, 1.28) |
| Chhattisgarh | 0.55***(0.39, 0.76) |
| Madhya Pradesh | 1.13 (0.76, 1.68) |
| Gujarat | 0.40***(0.29, 0.57) |
| Daman and Diu | 0.37***(0.24, 0.56) |
| Dadra and Nagar Haveli | 0.65*(0.44, 0.95) |
| Maharashtra | 0.98 (0.72, 1.33) |
| Andhra Pradesh | 0.85 (0.61, 1.17) |
| Karnataka | 0.63*(0.40, 0.96) |
| Goa | 0.65*(0.44, 0.96) |
| Lakshadweep | 0.60* (0.38, 0.94) |
| Kerala | 2.55***(1.78, 3.67) |
| Tamil Nadu | 4.28*** (3.09, 5.92) |
| Puducherry | 2.47***(1.75, 3.49) |
| Andaman and Nicobar Islands | 0.32*** (0.21, 0.51) |
| Telangana | 0.78 (0.56, 1.08) |
| **Note:** Dependent variable: Self Rated Health Conditions with three categories viz. Good, Fair and Poor. | |

| **Supplementary Table S5.** Binary logistic regression analysis of Work Limiting Health Conditions (N=23,951) among women in mid-life years (45-65 years), Longitudinal Ageing Study in India (LASI), wave-1, 2017-18. | |
| --- | --- |
| **Correlates** | **Adjusted Odds Ratio (95% C.I.)** |
| **Chronic Disease Score** |  |
| One (Single Disease) (Ref.) | 1.00 |
| Zero (None) | 0.68***(0.56, 0.83) |
| Two or more (Multimorbidity) | 1.42***(1.16, 1.75) |
| **Age (in years)** | 1.01* (1.00, 1.03) |
| **Residence** |  |
| Rural (Ref.) | 1.00 |
| Urban | 0.68***(0.54, 0.87) |
| **Religion** |  |
| Hindu (Ref.) | 1.00 |
| Muslim | 0.78(0.59, 1.04) |
| Christian | 1.39 (0.91, 2.12) |
| Others | 1.32(0.69, 2.51) |
| **Social Group** |  |
| Scheduled Castes (Ref.) | 1.00 |
| Scheduled Tribes | 0.67* (0.46, 0.94) |
| Other Backward Class | 1.01 (0.74, 1.39) |
| Other Castes | 1.12 (0.81,1.55) |
| **Level of education** |  |
| No Education (Ref.) | 1.00 |
| Less than Primary | 0.89 (0.63, 1.26) |
| Primary Completed | 1.03 (0.79, 1.34) |
| Middle Completed | 0.98 (0.69, 1.40) |
| Matric Completed | 0.64 (0.39, 1.05) |
| Intermediate Complete | 0.28***(0.11, 0.69) |
| Above Intermediate | 0.31***(0.15, 0.67) |
| **Occupation** |  |
| Unemployed (Ref.) | 1.00 |
| Blue Collar | 1.15(0.93, 1.42) |
| White Collar | 1.75 (0.92, 3.28) |
| Pink Collar | 1.37 (0.61, 3.08) |
| Not Classified/Others | 0.82 (0.59, 1.13) |
| **Wealth** |  |
| Poor (Ref.) | 1.00 |
| Middle | 1.08 (0.81, 1.85) |
| Rich | 1.01 (0.79, 1.28) |
| **Consumption of tobacco** |  |
| Tobacco Abstainer (Ref.) | 1.00 |
| Only Smoking | 1.22 (0.81, 1.85) |
| Only Smokeless | 1.05 (0.81, 1.35) |
| Both smoke and smokeless tobacco | 2.02 (0.66, 6.17) |
| **Ever use alcohol** |  |
| No (Ref.) | 1.00 |
| Yes | 1.22 (0.83, 1.78) |
| **Physical Activity** |  |
| Physically active (Ref.) | 1.00 |
| Physically inactive | 0.81* (0.65, 0.98) |
| **Waist-Hip Ratio** | 1.64 (0.51, 5.24) |
| **Marital Status** |  |
| Currently in Union (Ref.) | 1.00 |
| Not in Union | 1.22 (0.97, 1.52) |
| **Parity** | 1.02 (0.98, 1.06) |
| **History of Chronic Disease** |  |
| No (Ref.) | 1.00 |
| Yes | 1.12 (0.92, 1.35) |
| **Living Arrangements** |  |
| Living alone or with others (Ref.) | 1.00 |
| Living with family members | 0.89 (0.65, 1.22) |
| **Experienced Menopause** |  |
| No (Ref.) | 1.00 |
| Yes | 1.54* (1.04, 2.29) |
| Don't Know | 1.47 (0.89, 2.43) |
| **State** |  |
| Jammu and Kashmir (Ref.) | 1.00 |
| Himachal Pradesh | 0.38 (0.11, 1.23) |
| Punjab | 0.56 (0.17, 1.78) |
| Chandigarh | 0.33 (0.6, 1.81) |
| Uttarakhand | 1.05 (0.37, 2.99) |
| Haryana | 0.22*(0.06, 0.79) |
| Delhi | 0.73 (0.22, 2.39) |
| Rajasthan | 2.55 (0.99, 6.55) |
| Uttar Pradesh | 2.76* (1.08, 7.01) |
| Bihar | 1.67 (0.66, 4.24) |
| Arunachal Pradesh | 0.64 (0.12, 3.45) |
| Nagaland | 0.06*** (0.01, 0.44) |
| Mizoram | 2.07 (0.64, 6.67) |
| Tripura | 1.81 (0.64, 5.12) |
| Meghalaya | 1.10 (0.27, 4.43) |
| Assam | 1.09 (0.40, 2.99) |
| West Bengal | 0.58 (0.21, 1.62) |
| Jharkhand | 4.43*** (1.76, 11.17) |
| Odisha | 3.38* (1.32, 8.62) |
| Chhattisgarh | 0.88 (0.31 2.57) |
| Madhya Pradesh | 3.62**(1.40, 9.34) |
| Gujarat | 3.66*** (1.43, 9.40) |
| Daman and Diu | 2.53 (0.87, 27.03) |
| Dadra and Nagar Haveli | 10.23*** (3.87, 17.03) |
| Maharashtra | 0.56 (0.20, 1.56) |
| Andhra Pradesh | 4.16*** (1.65, 10.52) |
| Karnataka | 6.26***(2.32, 16.91) |
| Goa | 4.97** (1.88, 13.12) |
| Lakshadweep | 0.51 (0.13, 1.87) |
| Kerala | 0.67 (0.21, 2.09) |
| Tamil Nadu | 5.28***(2.06, 13.51) |
| Puducherry | 1.04 (0.36, 3.04) |
| Andaman and Nicobar Islands | 0.77 (0.18, 3.24) |
| Telangana | 3.71***(1.45, 9.47) |
| **Note:** Dependent variable: Work Limiting Health Conditions with two categories viz. At least One condition and None | |

| **Supplementary Table S6.** Binary logistic regression analysis of mobility restrictions (N=23,951) among women in mid-life years (45-65 years), Longitudinal Ageing Study in India (LASI), wave-1, 2017-18. | |
| --- | --- |
| **Correlates** | **Adjusted Odds Ratio (95% Confidence Interval)** |
| **Chronic Disease Score** |  |
| One (Single Disease) (Ref.) | 1.00 |
| Zero (None) | 0.54***(0.48, 0.61) |
| Two or more (Multimorbidity) | 1.85***(1.61, 2.13) |
| **Age (in years)** | 1.05***(1.61, 2.13) |
| **Residence** |  |
| Rural (Ref.) | 1.00 |
| Urban | 0.82*** (0.71, 0.93) |
| **Religion** |  |
| Hindu (Ref.) | 1.00 |
| Muslim | 1.34* (1.04, 1.62) |
| Christian | 0.85 (0.61, 1.20) |
| Others | 1.13 (0.83, 1.54) |
| **Social Group** |  |
| Scheduled Castes (Ref.) | 1.00 |
| Scheduled Tribes | 1.01 (0.82, 1.25) |
| Other Backward Class | 0.84* (0.74, 0.96) |
| Other Castes | 0.88 (0.76, 1.03) |
| **Level of education** |  |
| No Education (Ref.) | 1.00 |
| Less than Primary | 1.12 (0.95, 1.32) |
| Primary Completed | 1.20 (0.98, 1.47) |
| Middle Completed | 1.05 (0.86, 1.28) |
| Matric Completed | 1.02 (0.79, 1.32) |
| Intermediate Complete | 0.39***(0.24, 0.62) |
| Above Intermediate | 0.64* (0.41, 0.98) |
| **Occupation** |  |
| Unemployed (Ref.) | 1.00 |
| Blue Collar | 0.90 (0.79, 1.02) |
| White Collar | 0.96 (0.61, 1.51) |
| Pink Collar | 0.93 (0.66, 1.32) |
| Not Classified/Others | 0.84* (0.71, 0.98) |
| **Wealth** |  |
| Poor (Ref.) | 1.00 |
| Middle | 0.99 (0.87, 1.13) |
| Rich | 1.03 (0.91, 1.17) |
| **Consumption of tobacco** |  |
| Tobacco Abstainer (Ref.) | 1.00 |
| Only Smoking | 1.11 (0.89,1.39) |
| Only Smokeless | 1.08 (0.92, 1.26) |
| Both smoke and smokeless tobacco | 1.44 (0.75, 2.74) |
| **Ever use alcohol** |  |
| No (Ref.) | 1.00 |
| Yes | 0.99 (0.78, 1.28) |
| **Physical Activity** |  |
| Physically active (Ref.) | 1.00 |
| Physically inactive | 1.12*(1.01, 1.25) |
| **Waist-Hip Ratio** | 1.33 (0.68, 2.60) |
| **Marital Status** |  |
| Currently in Union (Ref.) | 1.00 |
| Not in Union | 1.19***(1.04, 1.36) |
| **Parity** | 1.01 (0.99, 1.04) |
| **History of Chronic Disease** |  |
| No (Ref.) | 1.00 |
| Yes | 1.32***(1.17, 1.47) |
| **Living Arrangements** |  |
| Living alone or with others | 1.00 |
| Living with family members | 1.01 (0.82, 1.25) |
| **Experienced Menopause** |  |
| No (Ref.) | 1.00 |
| Yes | 1.21* (1.02, 1.46) |
| Don't Know | 1.12 (0.88, 1.42) |
| **State** |  |
| Jammu and Kashmir (Ref.) | 1.00 |
| Himachal Pradesh | 0.84 (0.57, 1.23) |
| Punjab | 0.73 (0.49, 1.10) |
| Chandigarh | 0.89 (0.59, 1.33) |
| Uttarakhand | 1.00 (0.69, 1.44) |
| Haryana | 0.71 (0.50, 1.01) |
| Delhi | 0.80 (0.54, 1.18) |
| Rajasthan | 0.42*** (0.30, 0.59) |
| Uttar Pradesh | 0.96 (0.70, 1.32) |
| Bihar | 1.27 (0.91, 1.77) |
| Arunachal Pradesh | 0.34***(0.20, 0.57) |
| Nagaland | 0.58 (0.30, 1.14) |
| Mizoram | 0.36*** (0.21, 0.62) |
| Tripura | 0.72 (0.49, 1.05) |
| Meghalaya | 0.54* (0.33, 0.89) |
| Assam | 1.27 (0.90, 1.80) |
| West Bengal | 2.75*** (1.94, 3.91) |
| Jharkhand | 1.33 (0.95, 1.84) |
| Odisha | 1.00 (0.71, 1.42) |
| Chhattisgarh | 0.72 (0.50, 1.02) |
| Madhya Pradesh | 0.94 (0.65, 1.36) |
| Gujarat | 0.89 (0.63, 1.26) |
| Daman and Diu | 1.20 (0.79, 1.81) |
| Dadra and Nagar Haveli | 1.05 (0.70, 1.57) |
| Maharashtra | 1.80*** (1.29, 2.52) |
| Andhra Pradesh | 0.87 (0.61, 1.23) |
| Karnataka | 0.83 (0.52, 1.31) |
| Goa | 1.10 (0.75, 1.62) |
| Lakshadweep | 0.39***(0.25, 0.61) |
| Kerala | 1.54*(1.03, 2.31) |
| Tamil Nadu | 1.35 (0.95, 1.92) |
| Puducherry | 0.81 (0.54, 1.21) |
| Andaman and Nicobar Islands | 0.69 (0.45, 1.08) |
| Telangana | 0.59***(0.42, 0.83) |
| **Note:** Dependent variable: Mobility restrictions with two categories viz. Yes and No | |

| **Supplementary Table S7.** Zero-inflated Poisson regression analysis of Activities of daily Living (ADL) (N=23,951) among women in mid-life years (45-65 years), Longitudinal Ageing Study in India (LASI), wave-1, 2017-18. | |
| --- | --- |
| **Correlates** | **Prevalence Ratio (95 % Confidence Interval)** |
| **Chronic Disease Score** |  |
| One (Single Disease) (Ref.) | 1.00 |
| Zero (None) | 0.94 (0.88, 1.02) |
| Two or more (Multimorbidity) | 1.11*** (1.04, 1.17) |
| **Age (in years)** | 1.00 (0.99, 1.00) |
| **Residence** |  |
| Rural (Ref.) | 1.00 |
| Urban | 0.96 (0.90, 1.03) |
| **Religion** |  |
| Hindu (Ref.) | 1.00 |
| Muslim | 0.97 (0.89, 1.05) |
| Christian | 1.01 (0.87, 1.16) |
| Others | 1.04 (0.89, 1.23) |
| **Social Group** |  |
| Scheduled Castes (Ref.) | 1.00 |
| Scheduled Tribes | 1.07 (0.96, 1.20) |
| Other Backward Class | 1.01 (0.94, 1.09) |
| Other Castes | 1.04 (0.89, 1.22) |
| **Level of education** |  |
| No Education (Ref.) | 1.00 |
| Less than Primary | 0.89* (0.82, 0.97) |
| Primary Completed | 0.91*(0.84, 0.98) |
| Middle Completed | 0.85***(0.77, 0.94) |
| Matric Completed | 0.92 (0.81, 1.05) |
| Intermediate Complete | 0.83*(0.69, 0.99) |
| Above Intermediate | 0.90(0.76, 1.07) |
| **Occupation** |  |
| Unemployed (Ref.) | 1.00 |
| Blue Collar | 0.91*(0.85, 0.97) |
| White Collar | 0.92 (0.75, 1.12) |
| Pink Collar | 0.82*(0.69, 0.98) |
| Not Classified/Others | 0.88*(0.79, 0.98) |
| **Wealth** |  |
| Poor (Ref.) | 1.00 |
| Middle | 0.98 (0.91, 1.05) |
| Rich | 1.03 (0.97, 1.09) |
| **Consumption of tobacco** |  |
| Tobacco Abstainer (Ref.) | 1.00 |
| Only Smoking | 0.98 (0.85, 1.12) |
| Only Smokeless | 0.96(0.90, 1.04) |
| Both smoke and smokeless tobacco | 1.26 (0.89, 1.78) |
| **Ever use alcohol** |  |
| No (Ref.) | 1.00 |
| Yes | 1.00 (0.84, 1.19) |
| **Physical Activity** |  |
| Physically active (Ref.) | 1.00 |
| Physically inactive | 1.30***(1.22, 1.38) |
| **Waist-Hip Ratio** | 1.24 (0.87, 1.76) |
| **Marital Status** |  |
| Currently in Union (Ref.) | 1.00 |
| Not in Union | 0.99 (0.93, 1.06) |
| **Parity** | 1.01 (0.99, 1.02) |
|  |  |
| **History of Chronic Disease** |  |
| No (Ref.) | 1.00 |
| Yes | 0.93* (0.88, 0.98) |
| **Living Arrangements** |  |
| Living alone or with others (Ref.) | 1.00 |
| Living with family members | 0.94 (0.85, 1.02) |
| **Experienced Menopause** |  |
| No (Ref.) | 1.00 |
| Yes | 0.94 (0.84, 1.06) |
| Don't Know | 1.02 (0.87, 1.18) |
| **State** |  |
| Jammu and Kashmir | 1.00 |
| Himachal Pradesh | 0.91 (0.68, 1.23) |
| Punjab | 0.45***(0.33, 0.62) |
| Chandigarh | 0.53***(0.38, 0.73) |
| Uttarakhand | 0.76 (0.57, 1.02) |
| Haryana | 0.78 (0.53, 1.14) |
| Delhi | 0.67*(0.50, 0.91) |
| Rajasthan | 1.01 (0.68, 1.51) |
| Uttar Pradesh | 0.57***(0.44, 0.75) |
| Bihar | 0.58***(0.44, 0.74) |
| Arunachal Pradesh | 0.56* (0.32, 0.94) |
| Nagaland | 0.35***(0.26, 0.48) |
| Mizoram | 0.41***(0.28, 0.62) |
| Tripura | 0.77(0.55 1.07) |
| Meghalaya | 0.39***(0.26, 0.59) |
| Assam | 0.51***(0.38, 0.67) |
| West Bengal | 0.68***(0.52, 0.88) |
| Jharkhand | 0.49***(0.37, 0.65) |
| Odisha | 0.58***(0.43, 0.76) |
| Chhattisgarh | 0.70*(0.50, 0.97) |
| Madhya Pradesh | 0.71*(0.53, 0.95) |
| Gujarat | 0.73*(0.55, 0.97) |
| Daman and Diu | 0.64***(0.48, 0.86) |
| Dadra and Nagar Haveli | 0.54***(0.40, 0.74) |
| Maharashtra | 0.75*(0.58, 0.97) |
| Andhra Pradesh | 0.89(0.63, 1.24) |
| Karnataka | 0.67***(0.50, 0.91) |
| Goa | 0.61***(0.46, 0.82) |
| Lakshadweep | 0.66***(0.48, 0.89) |
| Kerala | 0.68***(0.52, 0.90) |
| Tamil Nadu | 0.68***(0.52, 0.90) |
| Puducherry | 0.64* (0.46, 0.90) |
| Andaman and Nicobar Islands | 0.73*(0.54, 0.98) |
| Telangana | 0.74 (0.52 1.04) |

| **Supplementary Table S8.** Zero-inflated Poisson regression analysis of Instrumental Activities of daily Living (IADL) (N=23,951) among women in mid-life years (45-65 years), Longitudinal Ageing Study in India (LASI), wave-1, 2017-18. | |
| --- | --- |
| **Correlates** | **Prevalence Ratio (95 % Confidence Interval)** |
| **Chronic Disease Score** |  |
| One (Single Disease) (Ref.) | 1.00 |
| Zero (None) | 0.91*** (0.87, 0.95) |
| Two or more (Multimorbidity) | 1.17***(1.12, 1.23) |
| **Age (in years)** | 1.02*** (1.01, 1.03) |
| **Residence** |  |
| Rural (Ref.) | 1.00 |
| Urban | 0.88*** (0.83, 0.92) |
| **Religion** |  |
| Hindu (Ref.) | 1.00 |
| Muslim | 0.98 (0.92, 1.04) |
| Christian | 1.10* (1.01, 1.21) |
| Others | 1.02 (0.90, 1.15) |
| **Social Group** |  |
| Scheduled Castes (Ref.) | 1.00 |
| Scheduled Tribes | 1.07 (0.99, 1.14) |
| Other Backward Class | 1.00 (0.95, 1.05) |
| Other Castes | 0.97 (0.92, 1.03) |
| **Level of education** |  |
| No Education (Ref.) | 1.00 |
| Less than Primary | 0.88*** (0.82, 0.94) |
| Primary Completed | 0.81*** (0.74, 0.86) |
| Middle Completed | 0.69***(0.62, 0.77) |
| Matric Completed | 0.59***(0.51, 0.68) |
| Intermediate Complete | 0.46*** (0.36, 0.58) |
| Above Intermediate | 0.32*** (0.25, 0.42) |
| **Occupation** |  |
| Unemployed (Ref.) | 1.00 |
| Blue Collar | 0.94* (0.90, 0.98) |
| White Collar | 0.74 (0.52 1.05) |
| Pink Collar | 0.81*** (0.70, 0.92) |
| Not Classified/Others | 0.84***(0.77, 0.91) |
| **Wealth** |  |
| Poor (Ref.) | 1.00 |
| Middle | 0.92*** (0.88, 0.96) |
| Rich | 0.94***(0.90, 0.98) |
| **Consumption of tobacco** |  |
| Tobacco Abstainer (Ref.) | 1.00 |
| Only Smoking | 1.07 (0.99, 1.16) |
| Only Smokeless | 1.05* (1.01, 1.11) |
| Both smoke and smokeless tobacco | 1.14 (0.95, 1.36) |
| **Ever use alcohol** |  |
| No (Ref.) | 1.00 |
| Yes | 1.00 (0.91, 1.09) |
| **Physical Activity** |  |
| Physically active (Ref.) | 1.00 |
| Physically inactive | 1.18*** (1.13, 1.23) |
| **Waist-Hip Ratio** | 0.84 (0.66 1.07) |
| **Marital Status** |  |
| Currently in Union (Ref.) | 1.00 |
| Not in Union | 1.03 (0.99, 1.08) |
| **Parity** | 1.00 (0.96, 1.04) |
| **History of Chronic Disease** |  |
| No (Ref.) | 1.00 |
| Yes | 1.00 (0.96, 1.04) |
| **Living Arrangements** |  |
| Living alone or with others (Ref.) | 1.00 |
| Living with family members | 1.00 (0.93, 1.08) |
| **Experienced Menopause** |  |
| No (Ref.) | 1.00 |
| Yes | 1.11* (1.02, 1.22) |
| Don't Know | 1.11 (0.99, 1.25 |
| **State** |  |
| Jammu and Kashmir (Ref.) | 1.00 |
| Himachal Pradesh | 0.73***(0.62, 0.85) |
| Punjab | 0.57*** (0.48, 0.68) |
| Chandigarh | 0.45*** (0.35, 0.58) |
| Uttarakhand | 0.99 (0.85, 1.14) |
| Haryana | 0.69*** (0.59, 0.80) |
| Delhi | 0.81 (0.65, 1.00) |
| Rajasthan | 0.63***(0.52, 0.76) |
| Uttar Pradesh | 0.84***(0.75, 0.94) |
| Bihar | 0.96 (0.86, 1.06) |
| Arunachal Pradesh | 0.30***(0.19, 0.47) |
| Nagaland | 0.19***(0.12, 0.30) |
| Mizoram | 0.12***(0.08, 0.19) |
| Tripura | 0.64***(0.54, 0.75) |
| Meghalaya | 0.33***(0.23, 0.48) |
| Assam | 0.98 (0.86, 1.11) |
| West Bengal | 0.87* (0.78, 0.96) |
| Jharkhand | 0.91 (0.80, 1.03) |
| Odisha | 0.46***(0.36, 0.58) |
| Chhattisgarh | 0.57*** (0.49, 0.67) |
| Madhya Pradesh | 0.84* (0.74, 0.96) |
| Gujarat | 0.73***(0.63, 0.84) |
| Daman and Diu | 0.54***(0.41, 0.69) |
| Dadra and Nagar Haveli | 0.76***(0.64, 0.90) |
| Maharashtra | 0.83 (0.68, 1.01) |
| Andhra Pradesh | 0.74***(0.66, 0.84) |
| Karnataka | 0.95 (0.85, 1.07) |
| Goa | 0.45***(0.35, 0.59) |
| Lakshadweep | 0.83 (0.68, 1.03) |
| Kerala | 0.71*** (0.59, 0.85) |
| Tamil Nadu | 0.91 (0.81, 1.02) |
| Puducherry | 0.59*** (0.50, 0.70) |
| Andaman and Nicobar Islands | 0.66*** (0.53, 0.83) |
| Telangana | 0.67***(0.59, 0.76) |

| **Supplementary Table S9**. Ordered logistic regression analysis of Life Satisfaction (N=23,951) among women in mid-life years (45-65 years), Longitudinal Ageing Study in India (LASI), wave-1, 2017-18. | |
| --- | --- |
| **Correlates** | **Adjusted Odds Ratio (95% Confidence Interval)** |
| **Chronic Disease Score** |  |
| One (Single Disease) (Ref.) | 1.00 |
| Zero (None) | 1.15***(1.04, 1.27) |
| Two or more (Multimorbidity) | 1.09 (0.98, 1.22) |
| **Age (in years)** | 0.98***(0.98, 0.99) |
| **Residence** |  |
| Rural (Ref.) | 1.00 |
| Urban | 0.96 (0.86, 1.00) |
| **Religion** |  |
| Hindu (Ref.) | 1.00 |
| Muslim | 1.02 (0.88, 1.19) |
| Christian | 1.16 (0.80, 1.69) |
| Others | 1.07 (0.85, 1.34) |
| **Social Group** |  |
| Scheduled Castes (Ref.) | 1.00 |
| Scheduled Tribes | 1.18* (1.01, 1.39) |
| Other Backward Class | 0.85***(0.75, 0.95) |
| Other Castes | 0.73***(0.64, 0.83) |
| **Level of education** |  |
| No Education (Ref.) | 1.00 |
| Less than Primary | 0.89(0.77, 1.01) |
| Primary Completed | 0.68***(0.59, 0.78) |
| Middle Completed | 0.56***(0.47, 0.66) |
| Matric Completed | 0.51***(0.39, 0.65) |
| Intermediate Complete | 1.00 (0.58, 1.71) |
| Above Intermediate | 0.56* (0.37 0.86) |
| **Occupation** |  |
| Unemployed (Ref.) | 1.00 |
| Blue Collar | 0.84*** (0.75, 0.93) |
| White Collar | 0.92 (0.51, 1.64) |
| Pink Collar | 1.11 (0.88, 1.39) |
| Not Classified/Others | 1.28***(1.10, 1.49) |
| **Wealth** |  |
| Poor (Ref.) | 1.00 |
| Middle | 091 (0.81, 1.02) |
| Rich | 0.84***(0.75, 0.93) |
| **Consumption of tobacco** |  |
| Tobacco Abstainer (Ref.) | 1.00 |
| Only Smoking | 1.27***(1.06, 1.53) |
| Only Smokeless | 1.18***(1.05, 1.33) |
| Both smoke and smokeless tobacco | 1.31 (0.83 2.06) |
| **Ever use alcohol** |  |
| No (Ref.) | 1.00 |
| Yes | 0.98(0.81, 1.18) |
| **Physical Activity** |  |
| Physically active (Ref.) | 1.00 |
| Physically inactive | 0.88* (0.80, 0.97) |
| **Waist-Hip Ratio** | 0.58 (0.33, 1.00) |
| **Marital Status** |  |
| Currently in Union (Ref.) | 1.00 |
| Not in Union | 1.42*** (1.28,1.57) |
| **Parity** | 0.99 (0.97, 1.41) |
| **History of Chronic Disease** |  |
| No (Ref.) | 1.00 |
| Yes | 1.28***(1.16, 1.42) |
| **Living Arrangements** |  |
| Living alone or with others (Ref.) | 1.00 |
| Living with family members | 0.64***(0.52 0.78) |
| **Experienced Menopause** |  |
| No (Ref.) | 1.00 |
| Yes | 1.17*(1.01, 1.36) |
| Don't Know | 1.29*(1.03,1.59) |
| **State** |  |
| Jammu and Kashmir (Ref.) | 1.00 |
| Himachal Pradesh | 0.22*** (0.16, 0.36) |
| Punjab | 0.35*** (0.26, 0.49) |
| Chandigarh | 0.29*** (0.22, 0.41) |
| Uttarakhand | 0.37***(0.27, 0.51) |
| Haryana | 0.48***(0.36, 0.63) |
| Delhi | 0.62***(0.45, 0.86) |
| Rajasthan | 0.76 (0.57, 1.01) |
| Uttar Pradesh | 0.69*** (0.53, 0.90) |
| Bihar | 0.45***(0.34, 0.59) |
| Arunachal Pradesh | 0.34***(0.21, 0.54) |
| Nagaland | 0.31***(0.15, 0.61) |
| Mizoram | 0.22***(0.14, 0.36) |
| Tripura | 0.38***(0.28, 0.52) |
| Meghalaya | 0.31***(0.20, 0.48) |
| Assam | 0.43***(0.33, 0.56) |
| West Bengal | 0.91(0.70, 1.17) |
| Jharkhand | 0.77 (0.58, 1.01) |
| Odisha | 0.60***(0.45, 0.79) |
| Chhattisgarh | 0.36***(0.27, 0.48) |
| Madhya Pradesh | 0.36***(0.27, 0.49) |
| Gujarat | 0.11***(0.08, 0.16) |
| Daman and Diu | 0.14***(0.09, 0.19) |
| Dadra and Nagar Haveli | 0.17***(0.11, 0.22) |
| Maharashtra | 0.26*** (0.19, 0.34) |
| Andhra Pradesh | 0.91 (0.68, 1.20) |
| Karnataka | 1.39 (0.92, 2.11) |
| Goa | 0.37***(0.28, 0.51) |
| Lakshadweep | 0.44***(0.29, 0.66) |
| Kerala | 0.76 (0.55, 1.07) |
| Tamil Nadu | 0.45***(0.34, 0.61) |
| Puducherry | 0.46***(0.34, 0.63) |
| Andaman and Nicobar Islands | 0.39*** (0.26, 0.61) |
| Telangana | 0.70* (0.53, 0.93) |
